# Synergistic effect of zinc oxide nanoparticles and antibiotics: a strategy to combat bacterial resistance

**DOI:** 10.1007/s44307-026-00137-4

**Published:** 2026-10-26

**Authors:** Mariana Picchi Salto, Natália Galvão de Freitas, Fabrício Costa Fanhani, Luana Mota Ferreira, João Augusto Oshiro Junior, Laurent Lemaire, Jean Leandro dos Santos, Leila Aparecida Chiavacci

**Affiliations:** 1 Pharmacy School, State University of São Paulo, Araraquara, SP Brazil; 2 Department of Pharmacy, Center of Health Sciences, Postgraduate Program in Pharmaceutical Sciences, Federal University of Paraná, Curitiba, PR Brazil; 3 Federal University of Paraiba, Postgraduate Program in Technological Development and Innovation in Medicines, João Pessoa, Paraíba Brazil; 4 Pharmaceutical Sciences Postgraduate Center for Biological and Health Sciences, State University of Paraíba, Campina Grande, Paraíba Brazil; 5 Univ Angers, INSERM, CNRS, MINT, SFR ICAT, Angers, F-49000 France; 6 Univ Angers, PRISM, SFR ICAT, Biogenouest, Angers, F-49000 France

**Keywords:** Zinc oxide nanoparticles, Antibacterial agents, Synergistic effects, Antimicrobial resistance, Nanotechnology

## Abstract

Antimicrobial resistance (AMR) continues to compromise the clinical utility of conventional antibiotics, highlighting the need for innovative adjuvant strategies. Zinc oxide nanoparticles (ZnO-NP) have gained attention due to their intrinsic antimicrobial properties and capacity to potentiate antibiotic activity through multimodal mechanisms. This scoping review systematically mapped current evidence on ZnO-NP with antibiotics combinations (ZnO-NP-antibiotic) following the PRISMA-ScR guidelines. Searches in PubMed, Scopus, and Web of Science identified 14 eligible studies. To ensure comprehensive coverage of the literature, an additional manual search was conducted using Google Scholar and the reference lists of the included studies, and 10 further articles were identified. In total, 24 articles are included in this scoping review. Most investigations relied on simple physical mixtures (n = 13); while fewer explored conjugation between ZnO-NP-antibiotic (n = 3); encapsulated systems (n = 4); or drug-loaded (n = 4). Better antimicrobial activity was consistently reported across multiple antibiotic classes. Although all studies evaluated planktonic activity, only 10 incorporated biofilm models. Notably, translational maturity remains limited: more than half of the studies (n = 14) did not include toxicity assessment, and safety evaluation was considered based on in vitro cytotoxicity assays (n = 5); in vivo investigations (n = 5) or Hen's Egg Test—Chorioallantoic Membrane (HET-CAM) (n = 1). These findings indicate that ZnO-NP-antibiotic systems remain predominantly in the early stages of development. Future progress will require standardized antibiofilm methodologies, systematic toxicological profiling, pharmacokinetic characterization, and mechanistic validation to establish reproducible structure–activity relationships. Addressing these translational and regulatory challenges will determine whether nanoparticle-mediated synergy can advance beyond a laboratory proof-of-concept toward clinically viable antimicrobial strategies.

**Supplementary Information:**

The online version contains supplementary material available at https://doi.org/10.1007/s44307-026-00137-4.

## Introduction

The discovery of penicillin marked a turning point in modern medicine, enabling the effective treatment of infectious diseases and leading to the development of multiple antibiotic classes (Saikia and Chetia 2024). However, decades of widespread and often inappropriate antibiotic use, combined with the limited development of new antimicrobial agents, have accelerated the emergence of AMR, which is now recognized as one of the most critical global health challenges of the 21st century (Saikia and Chetia 2024; Mahgoub et al. 2025; Masadeh et al. 2024; Pastore et al. 2025).

In this context, AMR represents a multifactorial and highly coordinated process involving structural defenses, genetic modifications, metabolic reprogramming, and cooperative survival strategies (Azeem et al. 2025; Li et al. 2020). Although primarily associated with genetic mutations and structural changes, new research indicates that some bacteria may possess a mechanism linked to adaptive phenotypic tolerance: certain highly resistant pathogens, such as *Staphylococcus aureus* (*S. aureus*), can actively respond to the electrostatic charge of a biomaterial by altering their own cell surfaces, thereby reducing the antimicrobial efficacy of the material with which the pathogen has come into contact (Hayles et al. 2025).

The contact between bacteria and a biomaterial creates an interface in which the efficacy of the biomaterial itself, in this case, the antibiotics, is dictated by the electrostatic properties of the environment. For instance, on negatively charged surfaces, bacteria can alter their own surface charge to become more positive and consequently repelling cationic antibiotics (such as vancomycin). This process allows the development of a tolerance of the treatments, as well as contributing to the formation of Biofilm communities (Hayles et al. 2025).

Biofilms are among the most clinically relevant manifestations of adaptive resistance. Their establishment and maintenance depend on a complex interaction of molecular and physiological determinants, including surface adhesion proteins, extracellular matrix biosynthesis, regulated gene expression, metabolic reprogramming, and collective stress responses (Masadeh et al. 2024; Azeem et al. 2025; Abdelghafar et al. 2022). The physical interface can act as the primary stimulus for the bacteria to initiate the transition from a planktonic state to a biofilm state. This process occurs through genetic regulation that alters the surface’s electrostatic charge, minimizing electrostatic repulsion and facilitating bacterial adhesion to surfaces, which enables biofilm development (Hayles et al. 2025).

Furthermore, this process also maintains the structural integrity of the biofilm, as bacteria utilize positively charged secretions to interact with anionic components of the cell surface, acting as “bridges” that reinforce the biofilm structure (Hayles et al. 2025). Given that AMR occurs because bacteria are not static targets, capable of actively modulating their biomolecular properties and extracellular matrix composition in response to the microenvironment (Hayles et al. 2025) to survive and to continue reproducing, it is highly necessary to identify mechanisms to combat this scenario.

In this context, nanotechnology emerges as a fundamental strategy to overcome the limitations of conventional therapies, specifically by using surface engineering to electrostatically regulate the nanoparticles, as well as improving the characteristics that the nanoparticles already provide. The use of compounds such as chitosan, for example, can adjust the nano system’s surface charge to maximize their affinity for the negatively charged membrane or to synchronize interactions with specific antibiotics, and neutralize the pathogen’s electrostatic defenses and phenotypic adaptations (Hayles et al. 2025; Bahadori Zade et al. 2024; Sathiyaseelan et al. 2023).

To address these challenges, ZnO-NP exhibit unique physicochemical properties. Their tunable size and morphology provide a high surface area-to-volume ratio, favoring close interaction with bacterial surfaces. This interaction is associated with multiple mechanisms (Mehta et al. 2019; Vasile et al. 2014; Pasquet et al. 2014), such as the release of Zn^2^⁺ ions, destabilization of the cell membrane, interference with metabolic pathways, and disruption of active transport systems (Masadeh et al. 2024).

**Fig. 1:**
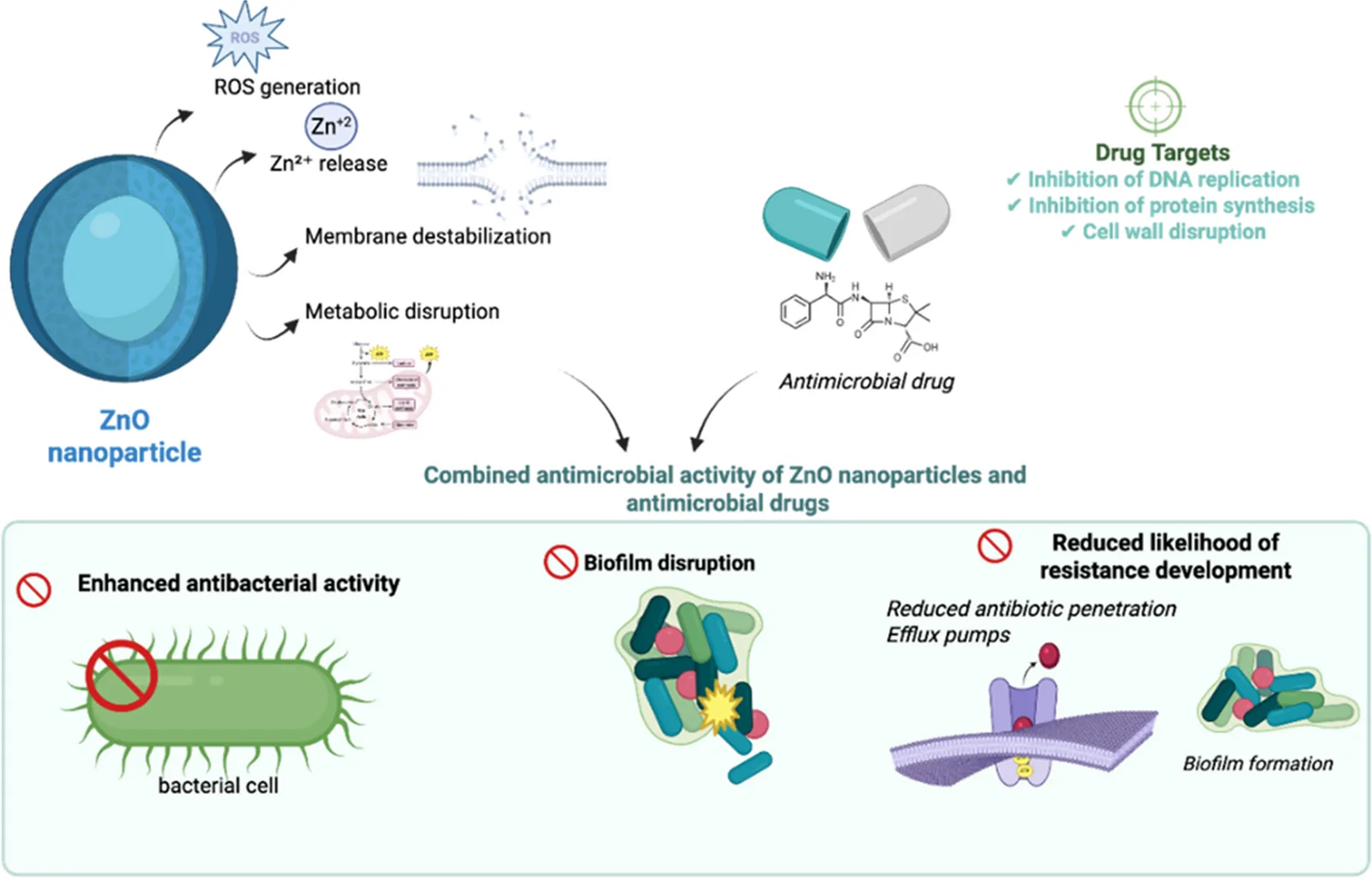
Conceptual representation of proposed mechanisms that may contribute to the enhanced antibacterial activity observed when ZnO-NP are combined with conventional antibiotics. Abbreviations: ROS: reactive oxygen species; Zn^2+^: zinc ions; ZnO: zinc oxide; DNA: deoxyribonucleic acid

Instead of acting on a single molecular target, ZnO-NP induce broad physiological stress that can affect multiple bacterial cell structures simultaneously. These multifaceted modes of action position nanomaterials as potential tools to complement existing drugs and mitigate the development of resistance (Masadeh et al. 2024; Elekhnawy et al. 2023; Hasannasab et al. 2021; Marchianò et al. 2020). Recent studies suggest that combining ZnO-NP with commercially available antibiotics may produce synergistic effects, increase antibacterial efficacy and contribute to delaying or overcoming antimicrobial resistance (Fadwa et al. 2021). An illustrative example is shown in Fig. 1.

As illustrated in Fig. 1, ZnO-NP can generate reactive oxygen species (ROS), release Zn^2^⁺ ions, destabilize bacterial membranes, and disrupt metabolic pathways. Concurrently, antibiotics target essential cellular processes such as deoxyribonucleic acid (DNA) replication, protein synthesis, and cell wall biosynthesis. The simultaneous occurrence and relative contributions of these mechanisms can enhance the antimicrobial efficacy of both agents. However, Fig. 1 should be interpreted as a conceptual framework of proposed complementary mechanisms rather than a universal model, as their combined effect may vary depending on the nanoparticles' size, morphology, surface chemistry, and aggregation state, as well as the antibiotic class, bacterial species, and experimental or environmental conditions.

In general, such systems are expected not only to enhance antimicrobial efficacy but also to optimize antibiotic delivery by improving drug retention at the infection site, enabling controlled or sustained release, maintaining effective therapeutic concentrations for prolonged periods, and reducing dosing frequency (Mahgoub et al. 2025; Ybarra et al. 2025; Gelen-Gungor et al. 2025; Seifi et al. 2024). These properties may ultimately improve patient adherence, decrease systemic exposure and mitigate risks associated with the overuse or inappropriate use of antibiotics (Mahgoub et al. 2025).

This scoping review systematically analyzes and integrates current evidence on ZnO-NP-antibiotic synergistic strategies, with emphasis on antimicrobial performance and translational limitations. By evaluating strategies based on experimental results, microbiological findings, and biological analyses, this work seeks to clarify the therapeutic potential of ZnO-NP-antibiotic combinations and identify important gaps and future directions in combating resistant bacterial strains.

## Methodology

The current scoping review was conducted according to the recommendations outlined by the Joanna Briggs Institute (Peters et al. 2015) and the results were reported according to the PRISMA Extension for Scoping Reviews (PRISMA-ScR) guidelines (Tricco et al. 2018). The review protocol was registered in the OSF database (DOI: https://doi.org/10.17605/OSF.IO/WA3Y7).

The review question and eligibility criteria were structured according to the PCC (Population, Concept, and Context) framework recommended by the Joanna Briggs Institute, in which the population comprised microorganisms evaluated in antimicrobial assays, the concept involved the ZnO-NP-antibiotic combinations, and the context corresponded to experimental microbiological studies assessing antimicrobial activity.

### Research strategy

The searches were conducted in November 2025 in three electronic databases: PubMed, Scopus, and Web of Science, with no restrictions on publication date or language. The same search strategy was applied across all platforms, using Boolean operators (“AND” and “OR”) to combine descriptors related to zinc oxide nanoparticles and antibiotics.

The search strategies were adapted to the syntax and indexing system of each database, when necessary. The complete strings used in PubMed, Scopus, and Web of Science, including database-specific field tags, Boolean operators, parentheses, and any applied filters, are provided in Supplementary Material, Table S1.

In PubMed, the MeSH terms and the Title/Abstract fields were used. In Scopus, the search was performed using article title, abstract, and keyword-based queries, while in Web of Science the Topic Search (TS) field was applied. In all cases, descriptors related to antimicrobial agents were combined with terms for zinc oxide nanoparticles using the Boolean operator AND, while synonyms within each group were combined using the OR operator.

To minimize the possibility of missing any eligible studies due to database-specific indexing or terminology, the database searches were complemented by manual screening of Google Scholar, as well as the reference lists of relevant articles. Additional eligible studies identified through these complementary approaches were screened according to the same predefined eligibility criteria.

### Eligibility criteria

Eligibility criteria were defined in accordance with the conceptual framework of this review, which focused on the antimicrobial activity of ZnO-NP and their potential synergistic interactions with antibiotics. Studies were included if they: (i) reported experimentally developed antimicrobial assays (e.g., microbiological evaluations such as minimum inhibitory concentration [MIC], minimum bactericidal concentration [MBC], disk diffusion, biofilm assays, or related methods), and (ii) investigated the synergistic effect between ZnO-NP-antibiotic, whether through simple co-administration, surface functionalization, chemical conjugation, or other forms of combined delivery.

Conversely, studies were excluded if they: (i) evaluated synergistic effects involving compounds other than antibiotics (e.g., metals or non-antibiotic materials), (ii) evaluated the combination of antibiotics and other metallic nanoparticles instead of ZnO-NP, or (iii) consisted of review articles, editorials, conference abstracts, book chapters, or other non-original research formats.

These criteria were established to address the central research question of this review: *What evidence exists regarding the antimicrobial effects of ZnO-NP combined with antibiotics?*

### Selection process

**Fig. 2:**
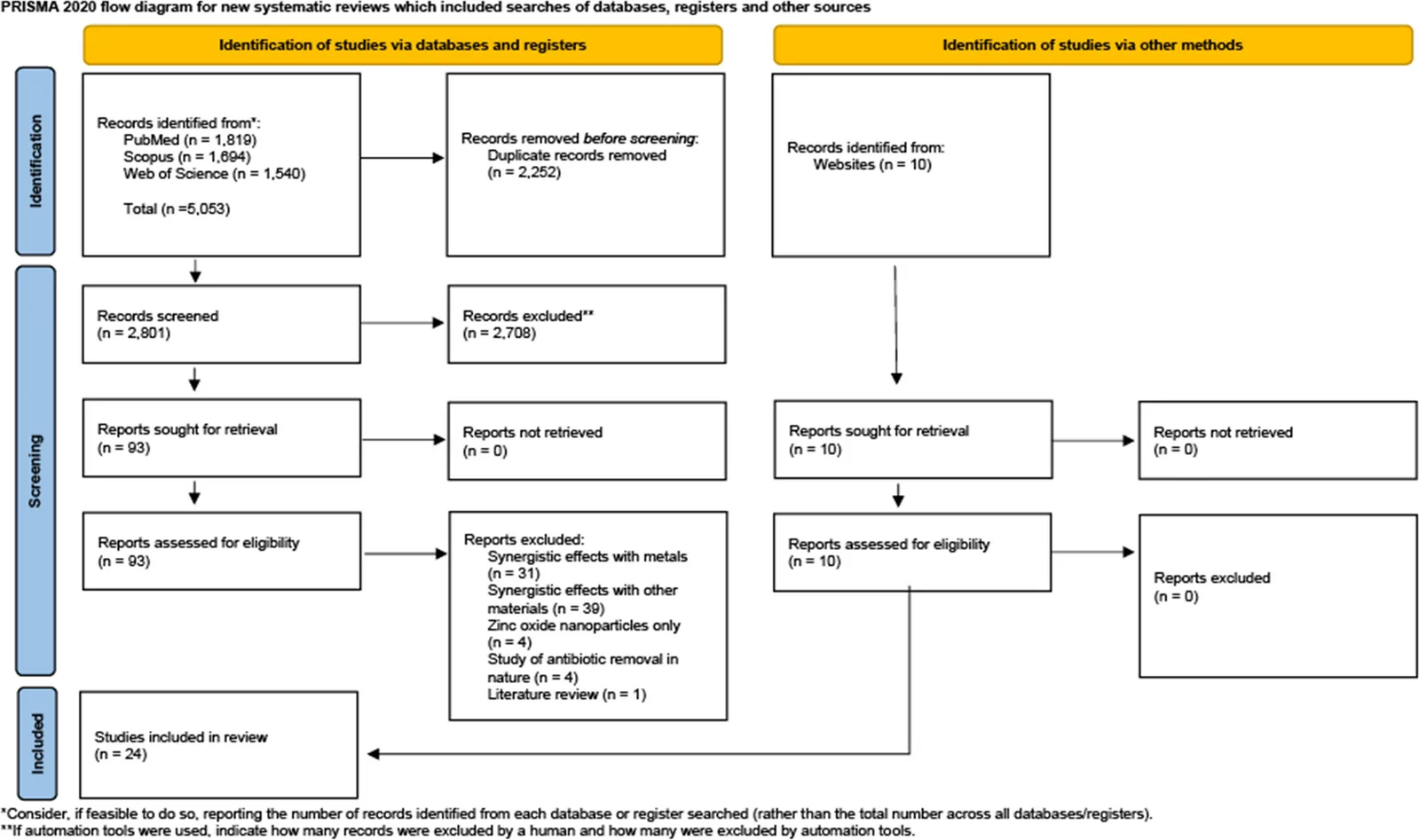
PRISMA-ScR flow diagram for scoping reviews illustrating the systematic screening, exclusion, and selection process of literature evaluating ZnO-NP-antibiotic combination. Abbreviations: PRISMA: Preferred Reporting Items for Systematic Reviews and Meta-Analyses; ZnO-NP-antibiotic: zinc oxide nanoparticles and antibiotics

To select articles, the researcher initially searched the databases using the described strategies. The results were compiled on the Rayyan web platform (free version; Rayyan Systems, Inc.) (Ouzzani et al. 2016) and duplicate articles were removed. Subsequently, two independent reviewers analyzed the titles and abstracts according to the inclusion criteria. Articles that met these criteria were submitted to full-text reviews to confirm eligibility. When there were disagreements about the inclusion of articles, the reviewers resolved the issue by reading the conflicting articles and then discussing them to reach a consensus. The excluded studies were recorded along with the specific reasons for exclusion (Supplementary Material, Table S2). The complete screening, exclusion, and selection process of the articles is illustrated in Fig. 2, a flowchart following the PRISMA-ScR standards.

### Data extraction and synthesis presentation

Data extraction was performed to collect all relevant information from the selected studies. For this purpose, the data were organized in Microsoft Excel® (Microsoft 365), containing the following information: title, authors, journal, publication date, ZnO-NP structural characteristics (size and morphology), whether there was functionalization with antibiotics, tested antibiotics, microbiological and toxicity assays performed, and specific antimicrobial outcomes.

The main results of each study were analyzed and discussed by all authors. The data and their respective categories were initially organized in Microsoft Excel®. Then, OriginPro® (64-bit, 2018) was used to create graphs, and Sankey diagrams were generated in Python using the Plotly library (version 5.18.0). This integrated approach provided a comprehensive synthesis of qualitative data, seeking to correlate research trends over time, identify the most frequently studied microorganisms, and highlight gaps for future research.

To improve the interpretation of antimicrobial synergy across heterogeneous studies, the evidence supporting synergistic or enhanced antimicrobial activity was further classified according to the methodological criteria used in each article. Studies reporting Fractional Inhibitory Concentration (FIC) or Fractional Inhibitory Concentration Index (FICI) values ≤ 0.5 and/or time-kill assay evidence (Masadeh et al. 2024; Abdelghafar et al. 2022; Fadwa et al. 2021; Sharma et al. 2016; El-Telbany et al. 2022; Masoudi et al. 2024; Khalil et al. 2025; Ravi et al. 2025) were categorized as presenting quantitatively supported synergy.

Studies reporting MIC/MBC reduction or increased inhibition zones without FIC/FICI or time-kill (Mahgoub et al. 2025; Fathi Azar Khavarani et al. 2016; Ghasemi and Jalal 2016; Thamer and Alsammak 2021; Saddik et al. 2022; Yu et al. 2024) were classified as potential synergy or enhanced antimicrobial activity. Studies reporting biofilm inhibition, reduction, or eradication without classical synergy criteria were classified as antibiofilm enhancement (Mohsenzadeh et al. 2025).

Finally, studies using the term “synergy” based mainly on statistical significance, qualitative comparison, enzyme inhibition, mechanistic endpoints, or non-standardized assays were classified as not quantitatively confirmed (Bahadori Zade et al. 2024; Sathiyaseelan et al. 2023; Murugesan et al. 2018; Anwar et al. 2020; Thakral et al. 2023; Iheme et al. 2024; Ibne Shoukani et al. 2024; Khuzaimah et al. 2025). This classification was used to support a more critical interpretation of the evidence and to avoid treating different experimental endpoints as equivalent indicators of true antimicrobial synergy.

### Methodological quality and risk of bias assessment

For studies involving in vivo animal experiments, the methodological quality was further evaluated using SYRCLE’s risk-of-bias tool tailored for animal research (Hooijmans et al. 2014). The assessment was organized in a table (Supplementary Material, Table S3) created with Microsoft Excel®. The evaluated domains included sequence generation, baseline characteristics, allocation concealment, random housing, blinding of caregivers and investigators, random outcome assessment, blinding of outcome assessors, incomplete outcome data, selective outcome reporting, and other bias sources (Hooijmans et al. 2014). Each domain was classified as “low risk,” “high risk,” or “unclear” based on the data reported in the original articles. If at least one domain was marked unclear and none were high risk, the overall bias classification was considered unclear. A study was deemed to have low overall bias only if all domains were rated low risk.

## Results

The process of study identification, encompassing database searches and manual retrieval, followed by screening and final inclusion, is presented in the PRISMA-ScR flow diagram for scoping reviews (Fig. 2).

The database searches yielded 5,053 records from PubMed, Scopus, and Web of Science. Of these, 2,252 were removed as duplicates, leaving 2,801 articles for title and abstract screening. After screening, 93 articles were considered potentially relevant and evaluated in full.

At this stage, the predefined inclusion and exclusion criteria were applied, resulting in the exclusion of 79 studies. The main reasons for exclusion were investigations addressing synergistic effects with metals (n = 31) or alternative materials (n = 39); studies evaluating ZnO-NP in the absence of association with antibiotics (n = 4); research focused on the removal of antibiotics from the environment (n = 4); and a review article (n = 1).

To broaden the sensitivity of the search and minimize the possibility of missing studies indexed under specific antibiotic names, the manual search also considered additional representative antibiotic terms and classes. This complementary step identified 10 further eligible studies, which were incorporated into the final analysis. As a result, 24 studies met all eligibility criteria and were included in the final qualitative analysis.

**Fig. 3:**
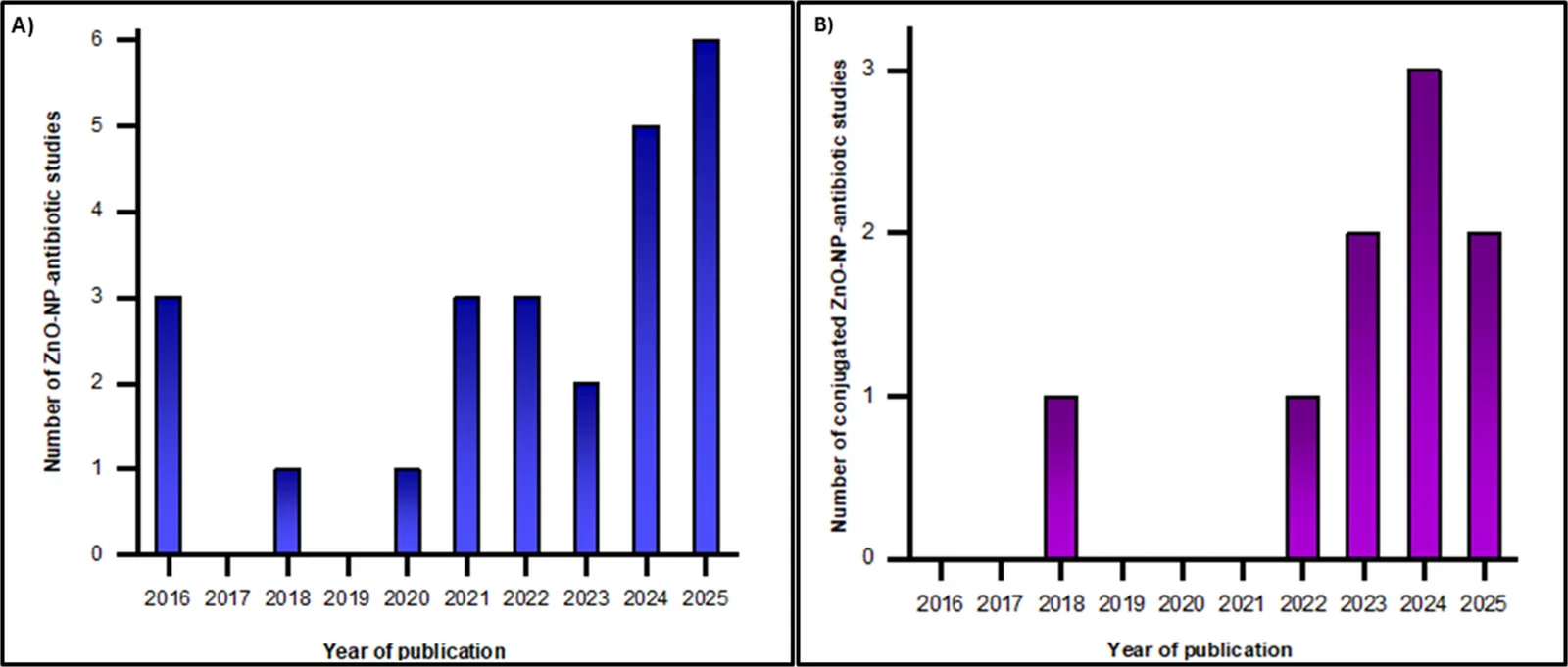
Annual distribution of publications on ZnO-NP-antibiotic systems from 2016 to 2025. **A** Total number of studies investigating combined antimicrobial applications (including physical mixtures, conjugation, encapsulation, and drug-loaded systems). **B** Subset of studies specifically reporting ZnO-NP chemically conjugated to conventional antibiotics over the same period. Abbreviations: ZnO-NP-antibiotic: zinc oxide nanoparticles and antibiotics

ZnO-NP have been widely investigated for their antimicrobial properties, as reflected by the large number of records initially retrieved (n = 5,053). However, studies specifically addressing their combined use with antibiotics remain relatively limited. Figure 3 provides a descriptive overview of the temporal distribution of the included studies published between 2016 and 2025.

As shown in Fig. 3A, studies considering ZnO-NP-antibiotic approaches were distributed across the last decade, with a higher number of publications in more recent years. Figure 3B shows that studies involving chemically conjugated ZnO-NP-antibiotic systems were less frequent than those based on simple co-administration, although they became more common from 2023 onward. This suggests a gradual shift from simpler experimental combinations toward more engineered nanostructured systems.

Regarding the biological aspects observed in the selected articles, it was possible to observe a difference between the bacterial strains studied as well as the biological tests developed and the antibiotics used also differed between the studies. Although the number of studies remains relatively limited, the selected articles reveal a notable diversity of experimental approaches and biological assays used to investigate the antimicrobial activity of ZnO-NP-antibiotic.

**Fig. 4:**
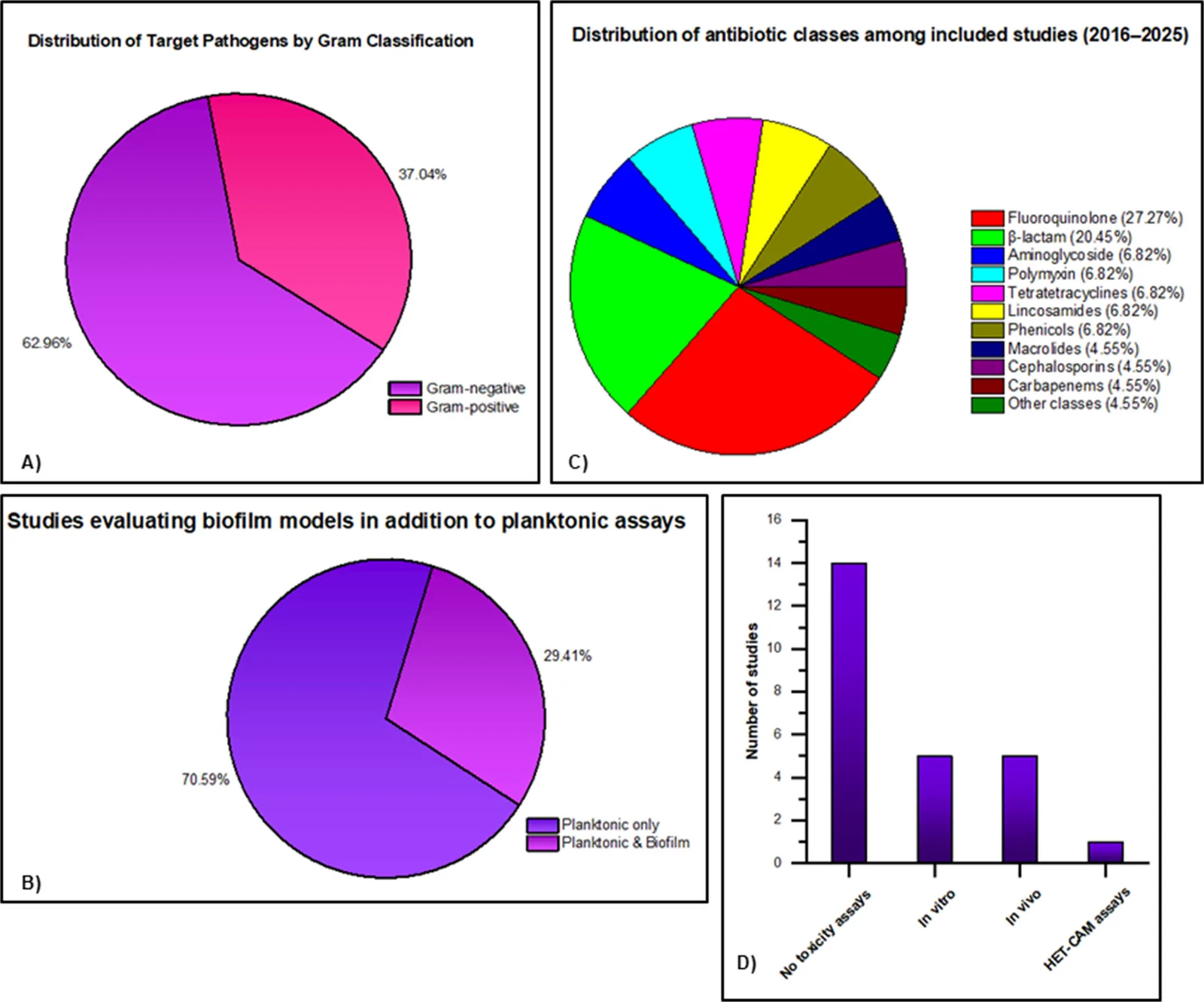
Summary of the main methodological and biological characteristics of the included studies mapping ZnO-NP-antibiotic combinations. **A** Percentage and distribution of the target microbial groups (Gram-negative versus Gram-positive bacterial pathogens); **B** Distribution of studies according to the experimental model employed (planktonic assays or planktonic assays combined with biofilm assays); **C** Frequency and distribution of conventional antibiotic classes investigated in combination with ZnO-NP; **D** Distribution of toxicity and safety assessment methods reported in the included studies. Abbreviations: ZnO-NP: zinc oxide nanoparticles; ZnO-NP-antibiotic: zinc oxide nanoparticles and antibiotics

The general characteristics of the selected studies, including the microbial groups investigated, the experimental conditions, the classes of antibiotics evaluated, and the toxicity assessments are summarized in Fig. 4:

In general, Fig. 4 highlights the studies observed in the selected articles. Initially, Fig. 4A shows that species of Gram-negative bacteria were the most frequently investigated (62.96%), compared to species of Gram-positive bacteria (37.04%). Although a few studies also reported the antimicrobial activity of ZnO-NP-antibiotic combinations against fungi and yeasts, these were not further discussed, as the scope of this review is restricted to bacterial pathogens and antimicrobial resistance.

Regarding experimental conditions, Fig. 4B shows that most studies evaluated synergistic effects only under planktonic conditions (58.3%). Some studies further supplemented this information by adding studies in biofilms, totaling 29.41%. Although planktonic assays remain the standard approach for initial screening, the considerable proportion of studies involving biofilms highlights a growing awareness of the clinical relevance of biofilm-mediated resistance.

Furthermore, regarding the distribution of antibiotic classes studied, Fig. 4C reveals that fluoroquinolones (27.27%) and β-lactams (20.45%) and were the most frequently explored combinations with ZnO-NP. Other classes, including tetracyclines, phenicol, lincosamides, aminoglycosides, polymyxins were reported less frequently.

Finally, the safety and toxicity assessments shown in Fig. 4D demonstrate that a substantial proportion of the studies did not include toxicity assessment (n = 14). Among those that performed safety analyses, in vitro, regarding cytotoxicity assays, were the most common (n = 5), followed by in vivo models (n = 5), and HET-CAM assays (n = 1). These results suggest that, although antimicrobial efficacy is widely investigated, comprehensive biocompatibility and translational assessments remain comparatively limited.

To provide a structured overview of the findings identified in the selected studies, Table 1 summarizes the main experimental parameters and results reported in each article, including whether ZnO-NP were conjugated to antibiotics or not, which classes of antibiotics were studied, the microbial targets, the synergistic evidence, and toxicity tests performed.

**Table 1:** Comparative summary of ZnO-NP-antibiotic formulations, experimental methodologies, and antimicrobial performance against target pathogens across the included studies (2016–2025)

Authors	Size ZnO-NP	Formulation type	Antibiotic class	Microorganisms	Antimicrobial analysis performed	Microbiology results: antibiotics	Microbiology results: ZnO-NP	Microbiology results: ZnO-NP-antibiotic	Synergism evidence	Mechanism proposal
Khuzaimah et al. (2025)	747 nm	Encapsulated system	Tetracycline, Nitroimidazole, β-lactam	*Pseudomonas aeruginosa (P. aeruginosa);* *Enterococcus faecalis (E. faecalis)*	Disk diffusion	Not rated	*P. aeruginosa:* 7.97 ± 0.49 mm *E. faecalis:* Absence of inhibition zone	*P. aeruginosa:* 8.76–11.27 mm *E. faecalis:* 2.00–7.50 mm	Statistical analysis shows an increase in the inhibition zone (p < 0.05)	Inhibition of cell wall formation, release of cytoplasmic content, and cell death
Ravi et al. (2025)	37–64 nm	Physical mixture	Polymyxin	*Klebsiella pneumoniae (K. pneumoniae)*	MIC	4–8 µg/mL	2–4 µg/mL	0.5–2 µg/mL	Fractional inhibitory concentration index (FICI) < 0.5	Membrane permeabilization, oxidative stress, and disruption of vital cellular functions
Mohsenzadeh et al. (2025)	Not rated	Physical mixture	Carbapenem	*P. aeruginosa*	MIC; Minimal biofilm eradication concentration (MBEC)	128–1024 µg/mL	128–1024 µg/mL	Reduction in *P. aeruginosa* biofilm formation	Statistical analysis shows an increase in the biofilm formation (p < 0.05)	Generation of ROS and the release of Zn^2+^ ions. Also genetically blocks their ability to form biofilms
Mahgoub et al. (2025)	Isolated: 72 nm Conjugated: 98 nm	Conjugated	Lincosamide	*Escherichia coli (E. coli); K. pneumoniae;* *Staphylococcus aureus (S. aureus);* *Listeria monocytogenes (L. monocytogenes)*	MIC	*E. coli:* 72.90 ± 27.57 µg/mL *K. pneumoniae:* 83.33 ± 20.83 µg/mL *S. aureus:* 104.17 ± 20.83 µg/mL *L. monocytogenes:* 104.17 ± 20.83 µg/mL	*E. coli:* 83.33 ± 20.83 µg/mL *K. pneumoniae:* 83.33 ± 20.83 µg/mL *S. aureus:* 83.33 ± 20.83 µg/mL *L. monocytogenes:* 83.33 ± 20.83 µg/mL	*E. coli:* 10.43 ± 2.63 µg/mL *K. pneumoniae:* 18.23 ± 6.87 µg/mL *S. aureus:* 18.23 ± 6.87 µg/mL *L. monocytogenes:* 10.43 ± 2.63 µg/mL	Statistical analysis shows a reduction in MIC values ​​for all strains tested, as well as an increase in the inhibition zone. (p < 0.05)	ZnO-NP increases the permeability of the bacterial cell membrane and generates ROS species. Clindamycin binds to the 50S ribosomal subunit, interrupting protein synthesis
Khalil et al. (2025)	7.6 ± 0.5 nm	Physical mixture	Lincosamide	*Bacillus* spp.	MIC; Inhibition of biofilms	*Bacillus licheniformis (B. licheniformis):* 64 µg/mL *Bacillus cereus (B. cereus):* 8 µg/mL *Bacillus thuringiensis (B. thuringiensis):* ≤ 128 µg/mL *Bacillus subtilis (B. subtilis):* ≥ 32 µg/mL *Bacillus megaterium (B. megaterium):* 2 µg/mL *Bacillus amyloliquefaciens (B. amyloliquefaciens):* ≥ 3 2 µg/mL	*B. licheniformis:* 32 µg/mL *B. cereus:* 4 µg/mL *B. thuringiensis:* ≤ 128 µg/mL *B. subtilis:* ≥ 32 µg/mL *B. megaterium:* 2 µg/mL *B. amyloliquefaciens:* ≥ 32 µg/mL	*B. licheniformis:* ≤ 16 µg/mL *B. cereus:* 1 µg/mL *B. thuringiensis:* ≤ 16 µg/mL *B. subtilis:* 8 µg/mL *B. megaterium:* ≤ 0.25 µg/mL *B. amyloliquefaciens:* 8 µg/mL	FICI *B. licheniformis:* 0.375 *B. cereus:* 0.375 *B. thuringiensis:* 0.75 *B. subtilis:* 0.5 *B. megaterium:* 0.5 *B. amyloliquefaciens:* 0.5	Generation of ROS. The attraction between the NP (positive) and the bacterial cells (negative) deforms the cell wall and disintegrates the membrane; ZnO-NP can disrupt the efflux pump system; The release of Zn^2+^ ions leads to genomic instability
Iheme et al. (2024)	99.02 nm	Conjugated	Fluoroquinolone	*S. aureus*	Half maximal inhibitory concentration (IC50)	Not rated	17.83 mg/mL	2.93 mg/mL	Statistical analysis shows a decrease in enzyme activity (p < 0.05)	Ciprofloxacin acts specifically on bacterial DNA gyrase and ZnO-NP exert intrinsic toxicity through the generation of ROS and damage to cell membrane integrity
Masadeh et al. (2024)	10 nm	Physical mixture	Fluoroquinolone, Polymyxin, Tetracycline, β-lactam	*E. coli* *S. aureus*	MIC, MBEC	*E. coli:* Norfloxacin (0.06 µg/mL), Colistin (0.1 µg/mL), Doxycycline (2 µg/mL) and Ampicillin (5500 µg/mL) *S. aureus:* Norfloxacin (38 µg/mL), Colistin (64 µg/mL), Doxycycline (0.125 µg/mL) and Ampicillin (30 µg/mL)	*E. coli:* 125 µg/mL *S. aureus:* 62.5 µg/mL Biofilms: 125 µg/mL	*E. coli:* Norfloxacin (0.06 µg/mL), Colistin (0.05 µg/mL), Doxycycline (2 µg/mL) and Ampicillin (2750 µg/mL) *S. aureus:* Norfloxacin (38 µg/mL), Colistin (16 µg/mL), Doxycycline (0.0625 µg/mL) and Ampicillin (15 µg/mL) Biofilms: 0.9765 µg/mL	*E. coli:* Norfloxacin (FICI: 3), Colistin (FICI: 0.531), Doxycycline (FICI: 3) and Ampicillin (FICI: 0.5156) *S. aureus:* Norfloxacin (FICI: 3), Colistin (FICI: 0.265), Doxycycline (FICI: 1.5) and Ampicillin (FICI: 0.5156) Biofilms (FICI: 0.0703)	Alteration of bacterial membrane integrity, increased cell permeability, which facilitates the influx, uptake, and intracellular accumulation of ZnO- NP, leading to the destruction of biological components (DNA, proteins, and lipids) through oxidative stress
Ibne Shoukani et al. (2024)	100–230 nm	Encapsulated system	Fluoroquinolone	*S. aureus; E. faecalis; E. coli; P. aeruginosa; K. pneumoniae* *Salmonella typhi (S. typhi)*	Disk diffusion; MIC; Minimum biofilm inhibitory concentration (MBIC)	From 2 mm (*E. coli*) to 9 mm (*S. typhi*)	From 11 to 15 mm	Inhibition zone 21 mm MIC 1.25 µg/mL (*S. aureus*; *S. typhi* and *E. faecalis*); 2.5 µg/mL (*E. coli*; *P. aeruginosa* and *K. pneumoniae*)	Statistical analysis shows better microbiological results and inhibition and destruction of biofilms (p < 0.05)	ZnO-NP causes the generation of ROS. The PEG coating facilitates deep penetration into the biofilm matrix and through the cell membrane, overcoming physical resistance barriers; Ciprofloxacin inhibits topoisomerase activity
Bahadori Zade et al. (2024)	Not rated	Encapsulated system	Aminoglycoside and cephalosporin	*P. aeruginosa; S. aureus;* *E. coli*	Disk diffusion	*P. aeruginosa:* 36 mm *S. aureus:* 30 mm *E. coli:* 34 mm	*P. aeruginosa:* 9 mm *S. aureus:* 11 mm *E. coli:* 13 mm	*P. aeruginosa:* 39 mm *S. aureus:* 35 mm *E. coli:* 35 mm	Statistical analysis shows better microbiological results (p < 0.05)	Generation of ROS. Chitosan potentiates this effect by interacting electrostatically with negative membranes. Antibiotics penetrate the cell and inhibit protein synthesis by reaching ribosomes
Masoudi et al. (2024)	57.87 nm	Physical mixture	β-lactam + β-lactamase inhibitor	*Acinetobacter baumannii (A. baumannii); E. coli*	MIC; Inhibition of biofilms	*A. baumannii:* 80 mg/mL *E. coli:* 1 mg/mL	4 mg/mL	*A. baumannii:* 1 mg/mL *E. coli:* 0.12 mg/mL	FICI ≤ 0.5	Disruption of the bacterial cell wall and membrane caused by ZnO-NP facilitates antibiotic entry into the cell; Generation of ROS; Release of Zn^2+^ ions
Yu et al. (2024)	35 nm	Drug-loaded nanoparticles	β-lactam and Fluoroquinolone	*P. aeruginosa; E. coli*	Growth kinetics in liquid medium	The antibiotics were ineffective when used alone	Minimal or no activity	Complete inhibition of *P. aeruginosa* growth within 24 h. Nearly twofold reduction in *E. coli* growth	Visual and statistical synergy demonstrated through growth kinetics assays (absorbance curves over 24 h)	Compromise the integrity of bacteria cell membranes and suppress the transcription of genes in bacteria that are resistant to oxidative stress
Sathiyaseelan et al. (2023)	Isolated: 40–60 nm Modified: 120–180 nm	Encapsulated system	Fluoroquinolone	*B. cereus; S. aureus; E. coli; Salmonella enterica (S. enterica)*	MIC; Inhibition of biofilms	*B. cereus:* 31.25–62.5 µg/mL *S. aureus:* 15.6–31.25 µg/mL *E. coli:* 15.6 µg/mL *S. enterica:* 7.8–15.6 µg/mL	From 125 to 250 µg/mL	*B. cereus:* 31.25 µg/mL *S. aureus:* 15.6 µg/mL *E. coli:* 7.8–15.6 µg/mL *S. enterica:* 3.9–7.8 µg/mL	Statistical analysis shows better microbiological results (p < 0.05)	Disruption of the integrity of the bacterial cell membrane; ZnO-NP can cause disruption of zinc homeostasis, which helps to break down resistance to beta-lactam antibiotics
Thakral et al. (2023)	33 nm	Conjugated	Fluoroquinolone	*E. coli; S. aureus;* *P. aeruginosa; E. faecalis*	Disk diffusion; Inhibition and eradication of biofilms	*E. coli:* 27.6 ± 2.51 mm *S. aureus:* 28.3 ± 1.15 mm *P. aeruginosa:* 29.6 ± 2.08 mm *E. faecalis:* 29 ± 2 mm	*E. coli:* 30 ± 1.73 mm *S. aureus:* 31 ± 2 mm *P. aeruginosa:* 30 ± 2.64 mm *E. faecalis:* 30 ± 1.73 mm	*E. coli:* 27.6 ± 2.51 mm *S. aureus:* 28.3 ± 1.15 mm *P. aeruginosa:* 29.6 ± 2.08 mm *E. faecalis:* 29 ± 2 mm	Statistical analysis shows better microbiological results and inhibition and destruction of biofilms (p < 0.05)	ZnO-NP can facilitate the antibiotic's reach to bacterial cells; in addition, levofloxacin exhibits advanced biocidal action
Abdelghafar et al. (2022)	50 nm	Physical mixture	Aminoglycoside, fluoroquinolone, β-lactam, lincosamide, macrolide, phenicol	*S. aureus*	MIC; MBIC; IC50	MIC Gentamicin: 4 µg/mL; Ciprofloxacin: 2 µg/mL; Chloramphenicol: 4 µg/mL; Cefotaxime: 128 µg/mL; Clindamycin: 0.03 µg/mL; Azithromycin: 0.5 µg/mL	IC50: 41.98 µg/mL Biofilm inhibition: 34%−37%	MIC Gentamicin: 0.25 µg/mL; Ciprofloxacin: 0.125 µg/mL; Chloramphenicol: 0.125 µg/mL; Cefotaxime: 4 µg/mL; Clindamycin: 0.003 µg/mL; Azithromycin: 0.125 µg/mL	FICI ≤ 0.5	Reduced adhesion capacity and biofilm formation, and induction of ROS that degrade cell wall proteins and disintegrate the membrane
El-Telbany et al. (2022)	Not rated	Physical mixture	Carbapenem	*P. aeruginosa*	Disk diffusion; MIC; Eradication of biofilms	512 µg/mL	64 µg/mL	4 µg/mL (ZnO-NP combined with meropenem); 8 µg/mL (meropenem combined with ZnO-NP); Complete eradication of pre-formed biofilm	FICI ≤ 0.5	While meropenem inhibits penicillin-binding proteins, weakening the peptidoglycan, ZnO-NP permeates the membrane and generates ROS that degrade proteins and DNA
Saddik et al. (2022)	39 nm	Drug-loaded nanoparticles	Macrolide	*E. coli; S. aureus*	Disk diffusion, MIC	Inhibition zone *E. coli:* 9,5 ± 2 mm *S. aureus:* 13 ± 3 mm MIC *E. coli:* 32 µg/mL *S. aureus:* 20 µg/mL	Inhibition zone *E. coli:* 2 mm *S. aureus:* 3 mm MIC *E. coli:* 55 µg/mL *S. aureus:* 30 µg/mL	Inhibition zone *E. coli:* 14 ± 3 mm *S. aureus:* 20 ± 4 mm MIC *E. coli:* 20 µg/mL *S. aureus:* 10 µg/mL	Synergy evaluated through nominal MIC reduction and inhibition zone increase	ZnO-NP can directly interfere with bacterial cell integrity, induce the release of Zn^+2^ ions, and generate ROS, which penetrate the bacterial membranes
Fadwa et al. (2021)	50 nm	Physical mixture	β-lactam, fluoroquinolone, polymyxin	*P. aeruginosa*	MIC	Colistin: 1 µg/mL; Meropenem: 16 µg/mL; Ciprofloxacin: 3.33 µg/mL;	From 2000 to 4000 µg/mL	From 0.33 to 1.33 µg/mL (colistin combined with ZnO-NP); 80 µg/mL (ZnO-NP combined with colistin)	FICI ≤ 0.5	Colistin increases the permeability of the outer membrane, which facilitates the accumulation of ZnO-NP inside the bacterial cell. The ZnO-NP generates oxidative stress, Furthermore, it is suggested that ZnO-NP may block efflux transporters, preventing the bacteria from expelling the antibiotic
Sharif et al. (2021)	Not rated	Physical mixture	Fluoroquinolone	*S. aureus*	Disk diffusion	Inhibition zone 0 mm	Inhibition zone 0 mm	Inhibition zone 7,70 ± 1,61 mm	Statistical analysis shows significant increase in the diameter of the inhibition zone (p < 0.001)	Generation of ROS and facilitating the antibiotic's action in inhibiting bacterial DNA gyrase
Thamer and Alsammak (2021)	20, 30 and 50–150 nm	Physical mixture	Glycopeptide	*S. aureus*	MIC	From 2500 to 5000 µg/mL;	From 5000 to 1000 0 µg/mL;	20 nm: from 39 to 78.125 µg/mL; 30 nm: from 39 to 78.125 µg/mL; 50–150 nm: from 39 to 156.25 µg/mL;	MIC results decreased in all sizes, but the 20 nm exhibited greatest inhibitory effect	ZnO-NP destroys the bacterial cell membrane and penetrates the cytoplasm. Generation of ROS, leading to the leakage of intracellular contents and cell death
Anwar et al. (2020)	20–40 nm	Drug-loaded nanoparticles	Aminoglycoside, phenicol	*E. coli*	Disk diffusion, MIC	MIC Gentamicin: 25.96 ± 8.95 µg/mL; Chloramphenicol: 52.04 ± 18.11 µg/mL Inhibition zone Gentamicin: 9.33 ± 0.5 mm; Chloramphenicol: 12.33 ± 0.57 mm	MIC 104,17 ± 36.08 µg/mL Inhibition zone 5.33 ± 0.58 mm	Both antibiotics coated MIC 10.42 ± 4.51 µg/mL Inhibition zone 22.00 ± 1.00 mm Gentamicin MIC 20.79 ± 8.95 µg/mL Inhibition zone 20.00 ± 1.00 mm Chloramphenicol MIC 25.96 ± 8,95 µg/mL Inhibition zone 17.00 ± 1.00 mm	Statistical analysis shows better microbiological results (p < 0.05)	ZnO-NP adheres to the outer layers of the bacteria, causing visible damage, pores in the cell wall, and subsequent leakage of cytoplasmic components. The combinations induce the filamentation process (where the cell grows but does not divide), which is an early indicator of irreversible bacterial damage
Murugesan et al. (2018)	Not rated	Conjugated	Fluoroquinolone	*B. cereus; P. aeruginosa*	MIC; minimum bactericidal concentration (MBC); Eradication of biofilms	MIC *B. cereus*: 55 µg/mL *P. aeruginosa:* 35 µg/mL MBC *B. cereus:* 80 µg/mL *P. aeruginosa:* 65 µg/mL	Not rated	MIC *B. cereus*: 25 µg/mL *P. aeruginosa:* 15 µg/mL MBC *B. cereus*: 60 µg/mL *P. aeruginosa:* 30 µg/mL	Not rated	The complex causes an antimotility effect, preventing bacteria from seeking nutrients and colonizing surfaces, which also reduces biofilms
Sharma et al. (2016)	20 nm	Physical mixture	Fluoroquinolone, β-lactam	*E. coli; K. pneumoniae;* *P. aeruginosa; S. typhi; S. aureus;* *B. subtilis*	MIC	MIC Ciprofloxacin: 0.5–4 µg/mL; Ampicillin: 1–34 µg/mL; Fluconazole: 0.12–16 µg/mL;	MIC From 0.16 to 0.43 mg/mL	Microbiology of ZnO-NP-antibiotic are not presented as absolute MIC values, but rather through Fractional inhibitory concentration (FIC)	10% Doped ZnO + Ciprofloxacin or with ampicillin showed synergism (100%, FIC ≤ 0.5) ZnO-NP-antibiotic: Majorly Additive (FIC > 0.5)	Increased ROS generation due to doping; electrostatic attraction with the bacterial cell wall
Ghasemi and Jalal (2016)	78 ± 7 nm	Physical mixture	Cephalosporin, fluoroquinolone	*A. baumannii*	Disk diffusion, MIC	Inhibition zone Ceftazidime: 0 mm Ciprofloxacin: 13 mm	MIC 0.5 mg/mL	Inhibition zone Ceftazidime: 33 mm Ciprofloxacin: 38 mm	Flow Cytometry Analysis demonstrated a pronounced increase in cell granularity, indicating greater internalization of antibacterial agents in the presence of ZnO-NP	The combination may form complexes that block efflux pumps, preventing the bacteria from expelling the antibiotic and allowing its intracellular accumulation
Fathi Azar Khavarani et al. (2016)	10–30 nm	Physical mixture	Quinolone, tetracycline, cephalosporin, phenicol, β-lactam	*Yersinia intermedia (Y. intermedia)*	Disk diffusion, MIC	Inhibition zone Oxacillin: 0 mm; Amoxicillin: 8 mm Ampicillin: 13 mm Chloramphenicol: 25 mm Ceftazidime: 25 mm Cefotaxime: 36 mm Tetracycline: 34 mm Nalidixic acid: 35 mm	MIC 39.06 µg/mL	Inhibition zone Oxacillin: 16 mm; Amoxicillin: 0 mm Ampicillin: 0 mm Chloramphenicol: 25 mm Ceftazidime: 25 mm Cefotaxime: 36 mm Tetracycline: 34 mm Nalidixic acid: 35 mm	Not rated	Adhesion of nanoparticles to the cell membrane, causing physical damage to the cell wall and generating oxidative stress

Note: Data are presented as reported in the original studies. All bacterial binomial names are highlighted in italics

*Abbreviations*: *P. aeruginosa Pseudomonas aeruginosa*, *E. faecalis Enterococcus faecalis K. pneumonia Klebsiella pneumonia*, *E. coli Escherichia coli*, *S. aureus Staphylococcus aureus*, *L. monocytogenes Listeria monocytogenes*, *Bacillus* spp. *Bacillus* multiple species, *B. licheniformis Bacillus licheniformis*, *B. cereus Bacillus cereus*, *B. thuringiensis Bacillus thuringiensis*, *B. subtilis Bacillus subtilis*, *B. megaterium Bacillus megaterium*, *B. amyloliquefaciens Bacillus amyloliquefaciens*, *S. typhi Salmonella typhi*, *A. baumannii Acinetobacter baumannii*, *S. enterica Salmonella enterica*, *Y. intermedia Yersinia intermedia*, *MIC* minimum inhibitory concentration, *MBEC* minimal biofilm eradication concentration, *IC50* Half maximal inhibitory concentration, *MBIC* minimum biofilm inhibitory concentration, *MBC* minimum bactericidal concentration, *ZnO-NP* zinc oxide nanoparticles, *ZnO-NP-antibiotic* zinc oxide nanoparticles combined with antibiotics, *FICI* fractional inhibitory concentration index, *FIC* fractional inhibitory concentration, *ROS* reactive oxygen species, *Zn*^*2+*^ zinc ions, *DNA* deoxyribonucleic acid, *PEG* polyethylene glycol

To address the methodological diversity found in the literature and standardize technical descriptions, the column *'formulation type'* was categorized into four groups based on the level of physicochemical integration between the components. The terms are defined as follows: *physical mixture*, referring to systems where ZnO-NP-antibiotic were co-administered separately to the test medium without prior association; *conjugated*, used when there was evidence of chemical bonding or surface functionalization between ZnO-NP-antibiotic, aimed to create a stable integrated entity; *encapsulated systems*, where both agents are embedded in a protective carrier matrix, such as chitosan or other polymers, to enhance stability and bioadhesion; and *drug-loaded nanoparticles*, referring to systems where the antibiotic was incorporated via physical adsorption on the surface or within the mesoporous structure of the ZnO-NP;

This compilation allows for a comparative analysis of the methodological approaches and antimicrobial performance among the studies. The data presented in this table serve as a basis for the subsequent discussion, which explores the reported antimicrobial activity, therapeutic implications, and current limitations.

**Fig. 5:**
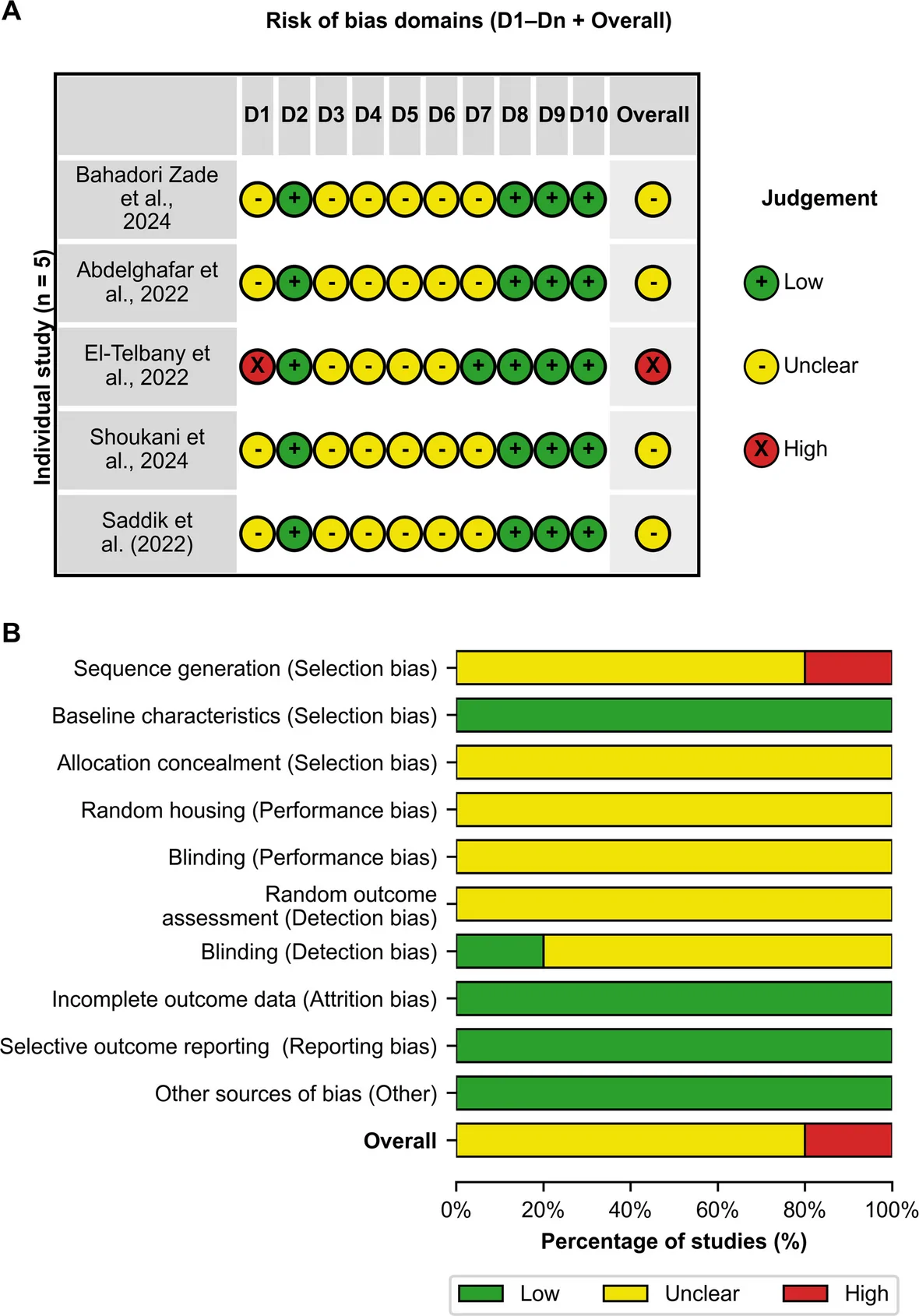
Risk-of-bias assessment of in vivo studies using SYRCLE’s tool. **A** Traffic-light plot showing the judgment for each risk-of-bias domain in the included in vivo studies. **B** Summary plot showing the percentage distribution of low, unclear, and high risk of bias across domains. D1: sequence generation; D2: baseline characteristics; D3: allocation concealment; D4: random housing; D5: blinding of caregivers/investigators; D6: random outcome assessment; D7: blinding of outcome assessors; D8: incomplete outcome data; D9: selective outcome reporting; D10: other sources of bias

The methodological quality of the in vivo studies was assessed using SYRCLE’s risk-of-bias tool, and the results are presented in Fig. 5. Five studies included in vivo animal experiments and were therefore evaluated across ten risk-of-bias domains.

As shown in Fig. 5A, most domains were classified as unclear risk of bias, particularly those related to sequence generation, allocation concealment, random housing, blinding of caregivers/investigators, and random outcome assessment. One study was classified as high risk for sequence generation. In contrast, baseline characteristics, incomplete outcome data, selective outcome reporting, and other sources of bias were generally classified as low risk. The overall judgment was unclear for all evaluated studies.

Figure 5B summarizes the percentage distribution of judgments across domains. Baseline characteristics, incomplete outcome data, selective outcome reporting, and other sources of bias were classified as low risk in all studies. However, several domains related to randomization, allocation, housing, and blinding were predominantly unclear. These methodological details are often underreported in in vivo experimental studies, but their absence limits the assessment of internal validity and reproducibility. Therefore, these findings should be interpreted as an indication of insufficient methodological reporting and as an alert for future studies to describe randomization, allocation concealment, housing conditions, blinding procedures, and outcome assessment more explicitly.

## Discussion

### Antibiotic classes and proposed complementary mechanisms

Antibiotics interfere with essential bacterial processes associated with survival, growth, and multiplication. Their therapeutic efficacy is closely linked to the structural and physiological characteristics of the target microorganisms, as differences in bacterial envelope organization, permeability, and metabolic state can directly influence both antibiotic access and the mechanism of action (Saikia and Chetia 2024). In this context, understanding how different classes of antibiotics interact with bacterial structures is particularly relevant when evaluating their combined use with ZnO-NP.

Some antibiotic classes require intracellular access to reach their targets (e.g., penicillin, cephalosporins, carbapenems, and monobactams, which belong to the β-lactam class, as they act similarly by inhibiting bacterial cell wall synthesis). Others act specifically on the bacterial cell membrane, such as polymyxins, which cause the disruption of this structure, that controls the influx and efflux of compounds and maintains cellular shape and structure (Saikia and Chetia 2024).

Conversely, ZnO-NP are usually described as antimicrobial agents with broad, multifactorial modes of action, rather than acting on a single molecular target (Khuzaimah et al. 2025). Their antimicrobial activity is commonly linked in the literature to physicochemical interactions with bacterial cells, including the generation of ROS, the release of Zn^2^⁺ ions, membrane destabilization, and the disruption of metabolic processes (Masadeh et al. 2024; Sharma et al. 2016; Khuzaimah et al. 2025).

It is suggested that the presence of ZnO-NP facilitates the action of antibiotics by increasing bacterial membrane permeability, promoting closer contact with the bacterial envelope, or contributing to the intracellular accumulation of antimicrobial agents. Some studies have also suggested possible effects on efflux pumps, which could reduce antibiotic export and increase intracellular drug concentrations (Masadeh et al. 2024; Abdelghafar et al. 2022; Sharma et al. 2016; Masoudi et al. 2024; Khalil et al. 2025; Ghasemi and Jalal 2016), while others have proposed that ZnO-NP can alter the integrity of the cell wall or bacterial membrane, causing structural damage that facilitates antibiotic entry (Sathiyaseelan et al. 2023; El-Telbany et al. 2022; Masoudi et al. 2024; Ghasemi and Jalal 2016; Anwar et al. 2020; Khuzaimah et al. 2025).

The coexistence of these proposed mechanisms offers a plausible explanation for the enhanced antimicrobial activity reported in various studies. However, in the context of combinations of ZnO-NP-antibiotic, these mechanisms should be interpreted with caution, since many of the included studies proposed these pathways based on the previously described behavior of ZnO-NP or on indirect antimicrobial results, rather than on direct mechanistic validation. Yet, the relative contribution of each pathway has not been sufficiently demonstrated in most of the included studies and should not be interpreted as universally confirmed for all systems combining ZnO-NP-antibiotic.

To facilitate the comparison of the antibiotic classes investigated, Table 2 summarizes the primary antimicrobial targets, the proposed complementary mechanisms for the combination with ZnO-NP, and the general qualitative results reported in the included studies. Although microbiological potential effects have been described for various antibiotic classes, direct comparisons should be also interpreted with caution, given that the studies differed considerably regarding bacterial strains, ZnO-NP physicochemical properties, formulation strategies, experimental conditions, and the methods used to assess antimicrobial activity. Consequently, the available evidence supports the potential of ZnO-NP-antibiotic combinations, encompassing distinct mechanisms of antibiotic action, but remains insufficient to determine whether any specific class of antibiotic consistently demonstrates superior antimicrobial performance.

**Table 2:** Comparative overview of antimicrobial classes investigated in ZnO-NP-antibiotic combinations

Antibiotic class	Number of included studies	Bacterial test occurrences	Main reported combined effects of ZnO-NP-antibiotic	Possible impact in combating AMR
Fluoroquinolone	12	Gram-positive (13) and Gram-negative (18)	Reduction of MIC, proposed inhibition of efflux proteins, and increased internalization of the antibiotic through membrane pores	High
β-lactam	8	Gram-positive (6) and Gram-negative (13)	Frequently associated with ROS generation, causing cell wall destruction, and reduction of biofilm	Promising
Aminoglycoside	3	Gram-positive (2) and Gram-negative (3)	Its polycationic nature facilitates binding to anionic bacterial surfaces, leading to irreversible cellular damage	Moderate
Polymyxin	3	Gram-positive (4) and Gram-negative (2)	Complementary disrupt the outer membrane with FIC values, suggests the entry of ZnO-NP, causing oxidative damage	High
Tetracyclines	3	Gram-positive (2) and Gram-negative (3)	Reported in encapsulated systems with improved antimicrobial activity and controlled release	Specific
Lincosamides	3	Gram-positive (5) and Gram-negative (2)	Reported high loading efficiency associating the inhibition of ribosomal protein synthesis	Specific
Phenicols	3	Gram-positive (1) and Gram-negative (2)	Reduction in MIC; when used as a coating, it induces physical damage to the outer layers of the bacteria	Specific
Macrolides	2	Gram-positive (2) and Gram-negative (1)	Increase in the zone of inhibition and significant acceleration of in vivo wound healing (95.8% wound closure)	Moderate
Cephalosporins	2	Gram-negative	Significant reduction in MIC; increased drug internalization and cellular granularity as evidenced by flow cytometry; increased zone of inhibition in resistant strains	High
Carbapenems	2	Gram-negative	Good results for the treatment and eradication of mature biofilms	Promising
Nitroimidazole	1	Gram-positive and Gram-negative	Effective inhibition of bacterial growth	Specific
Glycopeptide	1	Gram-positive	Reduction in MIC values; presented good response against multi-resistance bacterial strains	High

Note: The number of studies refers to the quantity of included articles in which each class of antibiotic was investigated; therefore, the total number of studies considering all antibiotic classes exceeds the total number of included studies, as more than one class of antibiotic was evaluated in some studies. Instances of bacterial testing represent the number of times Gram-positive or Gram-negative bacterial strains were investigated in the included studies. Thus, repeated tests of the same bacterial species or strain were counted as distinct instances. These counts should not be interpreted as the number of unique bacterial species

*Abbreviations*: *MIC* minimum inhibitory concentration, *ZnO-NP* zinc oxide nanoparticles, *ZnO-NP-antibiotic* zinc oxide nanoparticles combined with antibiotics, *FIC* fractional inhibitory concentration, *ROS* reactive oxygen species, *AMR* antimicrobial resistance

Table 2 presents a comparative overview of the antibiotic classes in ZnO-NP-antibiotic combinations, including the number of included studies reporting each class, the distribution of bacterial test occurrences according to Gram-positive or Gram-negative classification, the main antimicrobial effects reported, and their potential impact in combating AMR. The frequency of each antibiotic class reflects the number of studies in which it was investigated, for instance, a single study may contribute to more than one antibiotic class if multiple antibiotics are evaluated or if different bacterial strains are investigated.

Similarly, the counts of Gram-positive and Gram-negative bacteria correspond to the total number of strain occurrences in the selected studies, rather than the number of distinct bacterial species. Therefore, if the same bacterial species were evaluated in multiple studies, each occurrence was counted individually. This approach differs from the analysis presented in Figure 4A, in which each microbial species was counted only once, regardless of the number of studies in which it appeared.

Fluoroquinolones were the most frequently investigated class, being present in 12 studies, and it was evaluated against 13 Gram-positive and 18 Gram-negative bacterial strains. In other instances, such as with nitroimidazoles, the number of Gram-positive and Gram-negative strains was equal (only one of each); in others, such as with glycopeptides, research was conducted exclusively on Gram-positive strains or, as in the case of cephalosporins, exclusively on Gram-negative strains. Overall, Gram-negative bacteria predominated among the tested microorganisms, reflecting growing research interest in strategies capable of overcoming the intrinsic structural barriers and multidrug resistance commonly associated with this group.

It is important to note that the column regarding the potential impact of the antibiotic class on combating AMR encompasses results drawn from the studies themselves, although caution is advised when interpreting these data. Classes rated as “high”, such as fluoroquinolones, polymyxins, cephalosporins, and glycopeptides, consistently show reductions in MIC values when associated with complementary mechanisms of ZnO-NP-antibiotic systems.

In this regard, the authors frequently correlate these findings regarding the increased membrane permeability, enhanced intracellular antibiotic accumulation, possible efflux pump inhibition, and improved activity against multidrug-resistant strains (Masadeh et al. 2024; Ravi et al. 2025; Ghasemi and Jalal 2016; Thamer and Alsammak 2021). On the other hand, β-lactams and carbapenems were classified as “promising”, as available evidence highlighted particularly promising results against mature biofilms and multidrug-resistant pathogens, indicating a potentially significant role in addressing some of the most clinically challenging bacterial infections (El-Telbany et al. 2022; Masoudi et al. 2024; Mohsenzadeh et al. 2025).

Classes designated as specific, including tetracyclines, lincosamides, phenicols, and nitroimidazoles, were generally investigated within the scope of particular formulation strategies, such as encapsulated or coated delivery systems; consequently, available evidence remains restricted to specific experimental contexts, particularly in dental applications (Mahgoub et al. 2025; Khalil et al. 2025; Anwar et al. 2020; Khuzaimah et al. 2025). Similarly, aminoglycosides were classified as "moderate" because, despite promising results, including improved wound healing and antibacterial activity, current literature remains limited and is currently insufficient to draw broader conclusions regarding their applicability across different infection models (Bahadori Zade et al. 2024).

Overall, available evidence indicates enhanced antimicrobial activity across various antibiotic classes, suggesting that the benefits of ZnO-NP are not limited to a single mechanism of antibiotic action. However, the heterogeneity regarding nanoparticle characteristics, formulation strategies, bacterial species, and experimental methodologies currently precludes direct comparisons of synergistic performance among antibiotic classes. Future studies employing standardized protocols and quantitative synergy analyses will be essential to determine which specific antibiotic classes consistently yield the greatest benefit when combined with ZnO-NP.

### Influence of physicochemical properties on antimicrobial performance

The physicochemical characteristics of ZnO-NP are key determinants of their antimicrobial activity and directly influence their interaction with antibiotics, such as particle size (Masadeh et al. 2024; Masoudi et al. 2024; Thakral et al. 2023), surface charge (Masadeh et al. 2024; Sathiyaseelan et al. 2023; Ibne Shoukani et al. 2024), synthesis method (Sathiyaseelan et al. 2023; Ibne Shoukani et al. 2024), and surface functionalization (Masadeh et al. 2024; Sathiyaseelan et al. 2023; Ibne Shoukani et al. 2024). Among the included studies, particle size varied substantially, ranging from approximately 10 nm to several hundred nanometers, and this variation may affect surface area, bacterial membrane interaction, ROS generation, Zn^2^⁺ ion release, drug loading capacity, and cellular uptake (Masoudi et al. 2024).

In general, smaller nanoparticles are expected to provide a higher surface area-to-volume ratio, which may favor closer contact with bacterial envelopes and increase the density of reactive surface sites (Thakral et al. 2023). However, this relationship should be interpreted with caution because antimicrobial outcomes were evaluated using heterogeneous endpoints, including MICs, MBCs, inhibition zones, biofilm assays, and FICI values.

Beyond particle size, other physicochemical attributes such as morphology, surface charge, crystallinity, aggregation state, synthesis method, coating strategy, and surface functionalization may also modulate antimicrobial performance (Masadeh et al. 2024; Abdelghafar et al. 2022; Masoudi et al. 2024). Surface charge can influence electrostatic interactions with bacterial membranes (Hayles et al. 2025), while coatings such as PEG, chitosan, silica, or antibiotic functionalization may affect nanoparticle stability, dispersion, biocompatibility, drug loading, and release behavior (Mahgoub et al. 2025; Bahadori Zade et al. 2024; Sathiyaseelan et al. 2023; Anwar et al. 2020; Ibne Shoukani et al. 2024; Cao et al. 2023). Similarly, green synthesis methods may introduce bioactive residues or surface modifications that alter colloidal stability and biological activity (Masoudi et al. 2024; Iheme et al. 2024; Ibne Shoukani et al. 2024).

Despite their relevance, these physicochemical parameters were not uniformly reported across the included studies. Several articles provided limited information beyond particle size, and key parameters such as zeta potential, polydispersity, aggregation behavior in biological media, crystallinity, and surface chemistry were inconsistently described. This incomplete characterization limits direct comparison between studies and prevents the establishment of robust structure-activity relationships. Therefore, although available evidence indicates that the physicochemical properties of ZnO-NP contribute to antimicrobial enhancement, future studies should systematically report nanoparticle size distribution, morphology, zeta potential, crystallinity, aggregation state, surface functionalization, drug loading, and release profile under biologically relevant conditions.

### Interpretation of synergistic effects and methodological criteria

FICI is one of the most frequently used parameters to evaluate antimicrobial interactions in checkerboard assays. It is calculated from the fractional inhibitory concentration of each agent in combination relative to the MIC of the same agent when tested alone. Commonly used interpretative criteria consider FICI values ≤ 0.5 as indicative of synergy; values > 0.5 to 1.0 as additive; and values above 1.0 as indicative of indifferent or antagonism. However, these thresholds are not universally applied, and interpretative criteria may vary among studies (Gómara and Ramón-García 2019). Therefore, in the present review, FICI values were interpreted by considering the information from the original articles themselves. Yet, time-kill assays may also provide strong evidence of synergy, usually defined as a reduction of at least 2 log10 CFU/mL for the combination compared with the most active isolated agent (Fadwa et al. 2021; Gómara and Ramón-García 2019).

An important methodological aspect identified in this review was the heterogeneous use of the term “synergistic effect”. Although most included studies reported improved antimicrobial performance when ZnO-NP were combined with antibiotics, the criteria used to support this interpretation varied considerably. Some studies used quantitative interaction methods, such as FIC or FICI analyses, whereas others inferred synergistic behavior from increased inhibition zones, reductions in MIC, MBC, MBEC, antibiofilm effects, enzyme inhibition, or statistical significance alone. Since these approaches are not equivalent, the evidence supporting synergy was classified according to the methodological criteria used in each study, as summarized in Table 3. A detailed comparison of the main synergy assessment methods, including disk diffusion, MIC/MBC reduction, checkerboard/FICI analysis, time-kill assays, biofilm assays, and statistical comparison alone, is provided in Supplementary Material, Table S4.

**Table 3:** Classification of the methodological evidence supporting synergistic or enhanced antimicrobial activity in the included studies

Evidence category	Classification criteria	Interpretation adopted in this review	References
Quantitatively supported synergy	Studies reporting FICI ≤ 0.5 and/or time-kill assay evidence supporting synergism	Interpreted as stronger evidence of antimicrobial synergy, although still dependent on experimental design, tested concentrations, microbial strain, and reproducibility	(Masadeh et al. 2024; Abdelghafar et al. 2022; Fadwa et al. 2021; Sharma et al. 2016; Sharif et al. 2021; El-Telbany et al. 2022; Masoudi et al. 2024; Khalil et al. 2025; Ravi et al. 2025)
Potential synergy/enhanced antimicrobial activity	Studies reporting MIC/MBC reduction or increased inhibition zones, but without FICI, time-kill assay, or equivalent quantitative interaction analysis	Interpreted as enhanced antimicrobial activity or potential synergistic behavior, but not as definitive pharmacological synergy	(Mahgoub et al. 2025; Fathi Azar Khavarani et al. 2016; Ghasemi and Jalal 2016; Thamer and Alsammak 2021; Saddik et al. 2022; Yu et al. 2024)
Antibiofilm enhancement	Studies reporting inhibition, reduction, or eradication of biofilms, but without classical synergy criteria such as FICI or time-kill assays	Interpreted as evidence of improved antibiofilm performance, which may support therapeutic relevance but does not alone confirm antimicrobial synergy	(Mohsenzadeh et al. 2025)
Not quantitatively confirmed	Studies using the term “synergy” based mainly on statistical significance, qualitative comparison, enzyme inhibition, mechanistic endpoints, or non-standardized assays	Interpreted cautiously as supportive or exploratory evidence, requiring further standardized validation	(Bahadori Zade et al. 2024; Sathiyaseelan et al. 2023; Murugesan et al. 2018; Anwar et al. 2020; Thakral et al. 2023; Iheme et al. 2024; Ibne Shoukani et al. 2024; Khuzaimah et al. 2025)

Note: Classification was established based on the primary criteria reported in the origimal studies

*Abbreviations*: *FIC* fractional inhibitory concentration index, *MIC* minimum inhibitory concentration, *MBC* minimum bactericidal concentration

Based on this distinction, studies reporting FIC/FICI values or time-kill assays were interpreted in this review as providing stronger evidence of synergism. In contrast, studies based only on inhibition zone enlargement, MIC or MBC reduction, MBEC and antibiofilm improvement, enzyme inhibition, or statistical significance were interpreted more cautiously as evidence of enhanced antimicrobial activity or potential synergistic behavior, rather than definitive pharmacological synergy.

This distinction is particularly relevant because ZnO-NP-antibiotic systems may enhance antimicrobial performance through multiple mechanisms, including increased membrane permeability, oxidative stress, enhanced local exposure, improved drug delivery, or modulation of bacterial adhesion and biofilm formation. Nevertheless, these effects do not necessarily confirm synergism according to classical antimicrobial interaction models.

Therefore, although the current evidence supports the potential of ZnO-NP to enhance the antimicrobial activity of antibiotics, the interpretation of synergism remains limited by the absence of standardized criteria across studies. Future investigations should prioritize the systematic use of checkerboard assays, FICI calculations, time-kill curves, appropriate controls, resistant clinical isolates, and mechanistic validation. Such methodological standardization would strengthen the interpretation of ZnO-NP-antibiotic interactions and improve comparability across studies.

### Formulation strategies and reported antimicrobial interactions

To evaluate the presence of synergistic effects, the selected studies employed distinct experimental strategies combining ZnO-NP-antibiotic, categorized into four distinct groups: (i) Physical mixture; (ii) Conjugated; (iii) Encapsulated system; (iv) Drug-loaded nanoparticles. Each category and its main experimental approaches are discussed below.

#### Physical mixture

Most included studies (13 out of the 24 selected articles) investigated the synergistic effect through the simultaneous co-administration of ZnO-NP-antibiotic, without prior chemical bonding or structural integration (Masadeh et al. 2024; Abdelghafar et al. 2022; Fadwa et al. 2021; Sharma et al. 2016; Sharif et al. 2021; El-Telbany et al. 2022; Masoudi et al. 2024; Khalil et al. 2025; Ravi et al. 2025; Fathi Azar Khavarani et al. 2016; Ghasemi and Jalal 2016; Thamer and Alsammak 2021; Mohsenzadeh et al. 2025). Early investigations primarily adopted this approach, which remains widely used. In these cases, both agents were tested individually and in combinations.

For instance, some researchers (Abdelghafar et al. 2022) tested six different classes of antibiotics: aminoglycosides, fluoroquinolones, β-lactams, lincosamides, macrolides, and phenicols in *S. aureus* strains. The results showed improved antimicrobial results obtained for all classes, so that even with the worst results, it was still possible to observe a 60% reduction in bacterial biofilm (Abdelghafar et al. 2022).

In other studies, the aim was to verify whether the synergistic effect varied according to the microorganisms tested, rather than excessively varying the classes of antibiotics. It is the case of research in which (Sharma et al. 2016) two classes of antibiotics were tested, fluoroquinolones and β-lactams, in: *E. coli*, *K. pneumoniae*, *P. aeruginosa*, *S. typhi*, *S. aureus*, *B. subtilis*. The results showed that there was a reduction in the MIC value for all microorganisms tested in the presence of ciprofloxacin (fluoroquinolone) (Sharma et al. 2016).

In general, all studies were effective in demonstrating significant reductions in MIC, MBC values ​​and biofilm assays, suggesting that co-administration can be effective in promoting “synergistic effect”. However, only 5 studies (Abdelghafar et al. 2022; Sharma et al. 2016; El-Telbany et al. 2022; Masoudi et al. 2024; Ravi et al. 2025) conducted additional measurements of FIC or FICI index, methods considered essential to conclusively demonstrate pharmacological synergy. However, despite the results may indicate an enhancement of antimicrobial activity, this strategy alone does not guarantee that the antimicrobial results will be maintained in pharmaceutical formulations, highlighting the need for detailed release and pharmacokinetic studies.

#### Conjugated

A smaller subset (four articles) developed functionalized systems for ZnO-NP-antibiotic. In one study (Murugesan et al. 2018), ZnO-NP functionalized with humic acid and encapsulated ciprofloxacin achieved enhanced antimicrobial activity against *B. cereus* and *P. aeruginosa*, indicating success in the controlled interaction between the two antimicrobial agents. Furthermore, these results complemented controlled-release assays of the developed system over 24 h at different pHs, with the synergistic effect occurring in a targeted and controlled manner (Murugesan et al. 2018).

In another investigation, green-synthesized ZnO-NP facilitated levofloxacin delivery and enhanced bacterial activity (Thakral et al. 2023). The compound showed a potentiation of bacterial inhibition when compared to levofloxacin alone, suggesting that ZnO-NP could facilitate antibiotic penetration. The researchers also observed that the use of green nanotechnology could contribute to the stability of the compound in biological media, which may have allowed the antibiotic to access bacterial cells (Thakral et al. 2023).

Other research evaluated synergistic activity against *S. aureus* methicillin-resistant (MRSA) (Iheme et al. 2024). To this end, they developed ZnO-NP loaded with ciprofloxacin and evaluated their action by measuring the activity of the dehydrogenase enzyme, an essential biomarker for energy metabolism and bacterial viability. The authors were able to verify that at a concentration of 30 mg/mL, the developed compound achieved 100% inhibition of MRSA enzymatic activity. This efficacy could be attributed to the ability of the ZnO-NP to act as carriers that facilitate the access of ciprofloxacin to multiple molecular targets, enhancing the interruption of DNA synthesis and electron flow in the bacterial membrane (Iheme et al. 2024).

The synergistic potentiation of clindamycin in combination with ZnO-NP has also been explored (Mahgoub et al. 2025). Through physical adsorption onto the porous structure of the ZnO-NP, the antibiotic was bound, forming hydrogen bonds and electrostatic interactions between them. The complex showed significantly larger inhibition zones, as well as a reduction in MIC and MBC (Mahgoub et al. 2025). The good antimicrobial activity could be attributed to the ability of ZnO-NP to increase bacterial membrane permeability and generate ROS, facilitating the entry of clindamycin to inhibit protein synthesis in the ribosome (Mahgoub et al. 2025).

However, although studies have shown that the functionalization strategies employed can enhance therapeutic performance by increasing drug-loading capacity, none of the studies conducted complementary measurements to confirm the occurrence of synergistic effects. Most relied on statistical analysis, specifically ANOVA (Analysis of Variance) followed by multiple comparison tests (such as Tukey or Duncan), to demonstrate antimicrobial efficacy. These tests aimed to show that the combination of ZnO-NP-antibiotic was statistically superior to the use of either agent alone or to rule out the possibility that the results occurred purely by chance (Mahgoub et al. 2025; Thakral et al. 2023; Iheme et al. 2024).

While statistically superior results may indicate that the conjugation effectively enhanced antimicrobial activity, this method is not the standard for defining pharmacological synergy; the lack of data regarding the actual chemical interaction between the substances, as opposed to merely the statistical superiority of the final outcome, makes it difficult to confirm a synergistic effect (Gómara and Ramón-García 2019).

Finally, research has shown that the structural association between ZnO-NP-antibiotic can reduce premature drug loss and extend the duration of effective exposure at the infection site. However, long-term stability, reproducibility, and scalability in chemically functionalized systems remain underexplored, representing a significant translational gap.

#### Encapsulated system

Four recent studies integrated ZnO-NP-antibiotic into polymeric or hybrid matrices, with prominent applications in dentistry and wound healing. One study incorporated ZnO-NP into a mineral trioxide aggregate (MTA) matrix hydrated with chitosan and antibiotics (tetracycline, metronidazole, amoxicillin or ampicillin). Each compound was tested separately against strains of *P. aeruginosa* and *E. faecalis* (Khuzaimah et al. 2025).

For all antibiotics, an improved antimicrobial activity was shown, but tetracycline also showed the ability to increase the compressive and tensile strength of MTA, presenting promising characteristics for future applications. Compared to simple topical delivery systems, this chitosan-hydrated delivery model is designed to withstand mechanical loads in moist biological environments, such as the root canal (Khuzaimah et al. 2025).

The second study aimed to overcome resistance to ampicillin, in which the researchers (Sathiyaseelan et al. 2023) adopted a sophisticated, layered architecture approach, integrating the antibiotic into a chitosan matrix decorated with pegylated ZnO-NP. Initially, polyethylene glycol 3550 was added to the ZnO-NP to stabilize its morphology and reduce its tendency to agglomerate. Then, ampicillin was loaded onto pre-formed chitosan nanoparticles and finalized with the addition of ZnO-NP to its surface (Sathiyaseelan et al. 2023).

Antimicrobial tests showed a reduction in MIC and an increase in the inhibition zone for all microorganisms tested, in addition to demonstrating a superior ability to inhibit biofilm formation in *S. aureus* and *E. coli* when compared to free ampicillin. The differentiating factor of this methodology was the stratification of the nanocarrier, to isolate the ampicillin within the chitosan matrix before adding the ZnO-NP (Sathiyaseelan et al. 2023).

This technical choice was decisive, as the authors observed that direct loading resulted in the dissolution of ZnO-NP in an acidic medium and the inactivation of the antibiotic. The use of chitosan also made it possible to increase its biocompatibility, showing good results for HEK-293 cells and chicken embryos in HET-CAM assays (Sathiyaseelan et al. 2023).

The encapsulation technique was also successful to protect ZnO-NP from aggregation and reduce its cytotoxicity. In this reasarch, the nanoparticles were subjected to a reaction with polyethylene glycol to protect them with an outer layer, after this initial process, ciprofloxacin was then added to the ZnO-NP. The developed nano system showed low MIC values, biofilm inhibition, and excellent in vivo performance, indicating the eradication of infection in just two days, accelerating healing capacity by 20% (Ibne Shoukani et al. 2024).

Pegylation not only allowed for sustained and controlled release (77% to 90% in 12 h), but also made the nanoparticles biocompatible, with no evidence of hemolysis or significant cytotoxicity in BKK-21 (baby hamster kidney-21) cell lines. The mechanism of action could be attributed to the synergy between ROS generation by ZnO-NP and topoisomerase inhibition by ciprofloxacin, culminating in the massive leakage of bacterial proteins and DNA. This system can be characterized as an effective alternative to overcome fluoroquinolone resistance in severe skin infections (Ibne Shoukani et al. 2024).

The latest study regarding the development of an encapsulated system enabled research into an advanced nanofiber-based wound dressing for the combined delivery of two antibiotics and ZnO-NP (Bahadori Zade et al. 2024). The researchers employed a nanofiber system composed of a hybrid matrix of chitosan, polyvinylpyrrolidone, and gelatin, incorporating ZnO-NP and two antibiotics: amikacin (an aminoglycoside) and cefepime (a cephalosporine). Low doses of amikacin, because of its antimicrobial activity but higher toxicity, were used in combination with cefepime and ZnO-NP to assess the system's antimicrobial efficacy against hospital-acquired pathogens and to optimize it regarding systemic cytotoxicity (Bahadori Zade et al. 2024)

The results demonstrated larger zones of inhibition for the system compared to individual treatments against *P. aeruginosa*, *S. aureus*, and *E. coli*. Good cell viability (80%) was observed in cell L929, fibroblasts, alongside excellent compatibility with rabbit blood. Besides, significant acceleration in the healing of infected wounds was also observed over a 14-day period, with complete skin regeneration and new hair growth in groups treated with the complete system (Bahadori Zade et al. 2024).

In addition to presenting a more robust encapsulation methodology, these last two studies (Bahadori Zade et al. 2024; Ibne Shoukani et al. 2024) complemented their analyses with wound-healing assessments, indicating the potential of the developed compounds for treating skin infections. But, in general, all four studies related to encapsulated system also utilized statistical analysis to verify the antimicrobial efficacy of the compounds and their "synergistic effect." Despite the robustness of this research, measurements such as FIC and FICI are necessary to confirm the synergistic potential of the compounds; therefore, the findings should be interpreted cautiously as supportive or exploratory evidence requiring further standardized validation.

#### Drug-loaded nanoparticles

The final category concerns ZnO-NP loaded with antibiotics, specifically systems where the antibiotic was incorporated either via physical adsorption onto the surface or within the mesoporous structure of the ZnO-NP. The first study aimed to transform ZnO-NP into a drug-coating vehicle to modulate resistance in *E. coli* (Anwar et al. 2020). To this end, the ZnO-NP were coated with gentamicin, chloramphenicol, and both simultaneously, and subsequently tested against multidrug-resistant *E. coli* strains isolated from bovine milk samples associated with mastitis (Anwar et al. 2020).

The authors demonstrated that the dual-coated formulation yielded the best results, exhibiting the lowest MIC value and the largest zone of inhibition, representing a 312% increase in antimicrobial efficacy compared to ZnO-NP alone. It was also shown that using ZnO-NP as a vehicle ensured controlled release and improved drug solubility (Anwar et al. 2020).

Another study sought to develop a platform for treating infected wounds by adsorbing azithromycin onto the surface of ZnO-NP (Saddik et al. 2022). The nanocomposite was tested against MRSA and *E. coli* strains, resulting in a significant reduction in MIC values. Furthermore, the authors conducted in vivo experiments, finding that the group treated with the complete system achieved 95.8% wound healing within 10 days, demonstrating superior epidermal regeneration compared to control groups (Saddik et al. 2022). The authors proposed that the presence of zinc may have compromised bacterial cell membrane integrity and induced ROS production, while the azithromycin inhibited bacterial protein synthesis, enabling infection control even at lower drug dosages (Saddik et al. 2022).

Finally, the last study synthesized ZnO-NP with spherical, mesoporous morphological characteristics, enabling their use as a vehicle to carry and protect the antibiotic, thereby facilitating its penetration into bacterial cells (Yu et al. 2024). The authors found that the developed ZnO-NP exhibited high loading capacity and biocompatibility, while also overcoming limitations associated with conventional therapies, such as low bioavailability and short half-life.

Accordingly, mesoporous ZnO-NP were designed for the protected delivery of piperacillin-tazobactam and ciprofloxacin and subsequently tested against *E. coli* and multidrug-resistant *P. aeruginosa.* It was observed that the agents alone, whether ZnO-NP or antibiotics, failed to inhibit *P. aeruginosa* growth, whereas the combination achieved absolute bactericidal efficacy after 24 h of growth kinetics monitoring. Furthermore, the mesoporous structure acted as an "extraordinary therapeutic nanocarrier," enhancing the antibiotic's biodistribution and stability in the culture medium and facilitating its penetration across the bacterial lipid barrier through oxidative stress (Yu et al. 2024).

The three studies differed in their approaches to measuring and classifying the synergistic effect. In the first study, in addition to evaluating the antimicrobial response of the antibiotics alone and in combination with ZnO-NP, the researchers examined bacterial filamentation as a biomarker of synergistic damage (Anwar et al. 2020). Microscopic observation of the bacterial cells following treatment with the developed compound revealed that, although the antimicrobial treatment allowed for bacterial growth, it effectively inhibited cell division, a characteristic the authors classified as an early indicator of irreversible bacterial damage. Furthermore, statistical analyses were used to confirm antimicrobial efficacy, yielding values ​​consistent with potential synergy (Anwar et al. 2020).

In the second study, synergism was assessed based on a reduction in MIC values ​​and a significant increase in zones of inhibition compared to the compounds used individually (Saddik et al. 2022). The third study employed a different approach; prioritizing growth kinetics monitored via optical density (OD at 600 nm) over a 24-h period (Yu et al. 2024). This method assesses how a bacterial population multiplies over time, providing a dynamic profile of the microorganism's response to treatment.

The results showed that bacteria treated with either antibiotics alone or ZnO-NP alone exhibited rising growth curves, indicating resistance, whereas those treated with the ZnO-NP-antibiotic combination showed results close to zero, visually and statistically confirming the treatment's efficacy (Yu et al. 2024). The advantage of this treatment lies in the ability to identify not only whether growth was inhibited but also the specific point in time when this occurred; however, it has the limitation of not distinguishing between cells that are alive but not growing and those that have actually been killed (Yu et al. 2024).

Regarding the drug-loaded experiments, it is important to note that, although all three addressed "synergistic" microbiological results, the FIC or FICI index was not calculated; instead, a direct performance comparison was made between the agents used in isolation and in combination, leading to their classification as exhibiting "potential synergism" throughout this research.

### Microbiological profile and biological tests

All selected studies primarily used planktonic antimicrobial assays, specifically MIC, MBC, and zone of inhibition. These analyses represent an essential first step in antimicrobial screening, providing standardized and reproducible measures of bacterial growth inhibition. However, these models do not fully replicate the complex microenvironment found during clinical infections, making biofilm studies necessary, since in vivo many bacterial infections are characterized by biofilm formation rather than free-living bacterial populations (Masadeh et al. 2024; Abdelghafar et al. 2022; Murugesan et al. 2018).

Some pathogens possess biological system that modifies the cell surface to make it less negative. The adhesion to surfaces influences the expression of specific genes, which can alter the cell charge and create a barrier against positively charged antibiotics (Hayles et al. 2025). This is particularly relevant to biofilms, because they are reinforced by “electrostatic bridges”, where positively charged bacterial secretions interact with anionic cell surface components to maintain matrix cohesion (Hayles et al. 2025). Consequently, this process creates a high tolerance to antibiotics, demonstrating that biofilm-associated resistance is mediated by electrostatic interactions. Biofilm formation is not merely a physical barrier, but a dynamic process of electrostatic repulsion that protects the bacterial colony from specific drugs (Hayles et al. 2025).

**Fig. 6:**
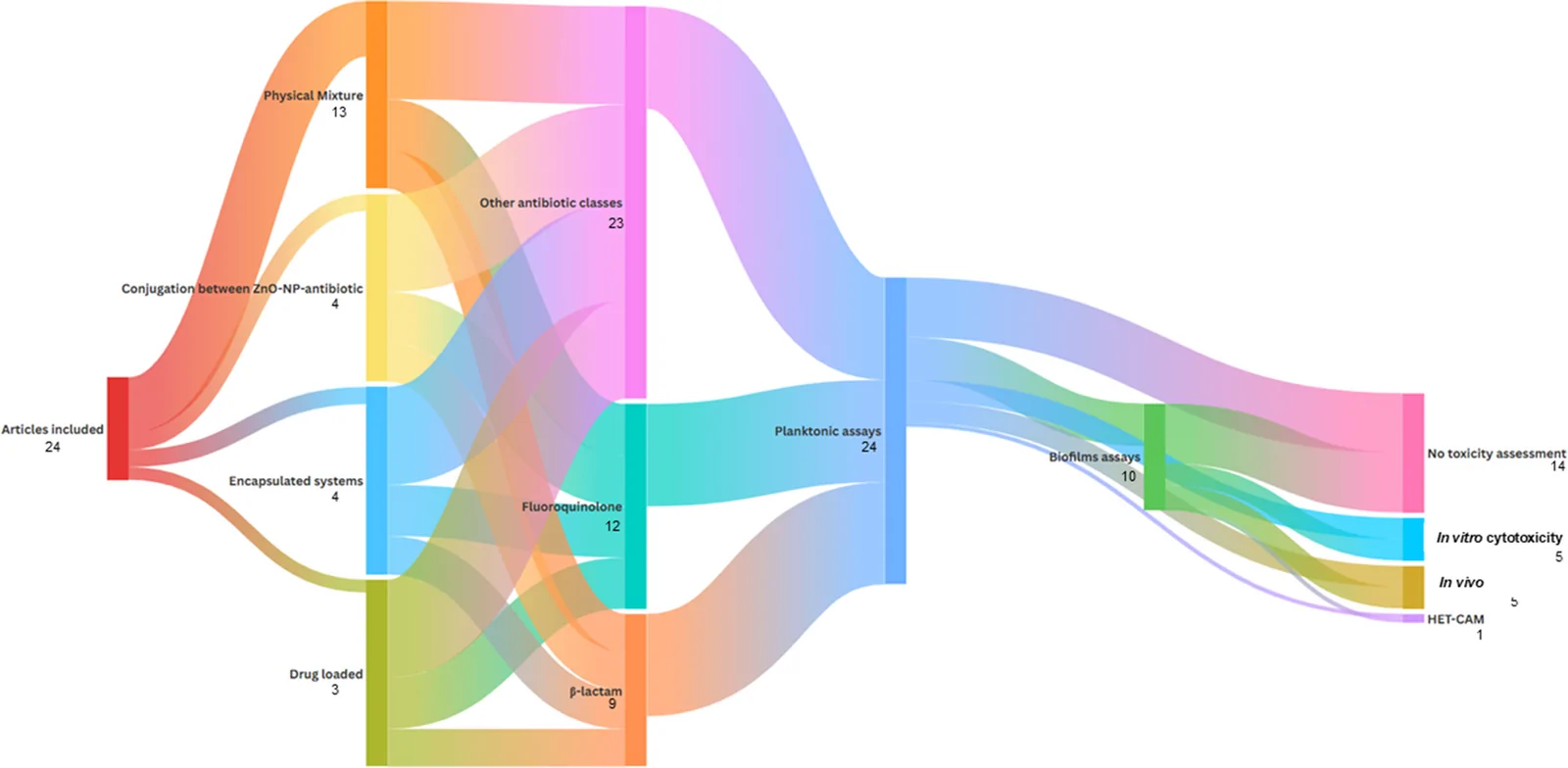
Sankey flow diagram mapping the methodological trajectory and evidence distribution of included studies (n = 24) evaluating ZnO-NP-antibiotic combinations. Caption: Flow diagram illustrating the progression of reported research from formulation type (*physical mixture, conjugated systems, encapsulated systems, and drug-loaded nanoparticles*) to target antibiotic classes (*fluoroquinolones, β-lactams, and other antibiotic classes*), experimental antimicrobial models (*planktonic assays versus complementary biofilm assays*), and biological safety/toxicity evaluations (*no toxicity assessment, *in vitro* cytotoxicity assays, *in vivo* models, and HET-CAM assay*). Note: Node heights and flow line widths are directly proportional to the frequency of included studies. Abbreviations: HET-CAM: Hen's Egg Test Chorioallantoic Membrane

As a result, biofilm formation is associated with persistent infections and increased AMR, so evaluating the activity of antimicrobial systems against biofilms is particularly relevant when assessing their therapeutic potential (Li et al. 2025). Of the 24 selected articles, 10 expanded their antimicrobial assays to include tests on bacterial biofilms (Masadeh et al. 2024; Abdelghafar et al. 2022; Sathiyaseelan et al. 2023; El-Telbany et al. 2022; Masoudi et al. 2024; Khalil et al. 2025; Mohsenzadeh et al. 2025; Murugesan et al. 2018; Thakral et al. 2023; Ibne Shoukani et al. 2024). To illustrate, Fig. 6 provides correlations between the antimicrobial assays.

As shown in Fig. 6, given that biofilms represent a major clinical barrier in combating AMR, the low representation of these tests suggests that much of the research remains at an early proof-of-concept stage. Planktonic, regardless of the antibiotic class, were conducted in a standardized manner across studies, unlike biofilm assays, underscoring the need for standardized antibiofilm methodologies and more systematic toxicological profiling to enable these systems to advance toward real-world clinical applications.

Most studies used the 96-well microplate assay with crystal violet staining to quantify biofilm biomass. In these experiments, bacteria were cultured in liquid medium, allowing them to adhere to the walls and bottoms of the plate wells and form a biofilm. Following an established adhesion period, it was possible to test multiple concentrations of nanoparticles and antibiotics simultaneously within a compact space.

Based on this, inhibition and eradication tests were conducted, representing two distinct approaches to evaluating the efficacy of treatments against bacterial biofilm communities. In inhibition tests, the compounds were added to the culture medium simultaneously with the bacterial inoculum to assess their ability to prevent biofilm formation (Sathiyaseelan et al. 2023; Masoudi et al. 2024; Khalil et al. 2025; Ibne Shoukani et al. 2024). Conversely, some studies also performed biofilm eradication tests to determine whether the developed compounds could penetrate the existing bacterial matrix, kill protected cells, and/or disintegrate the biofilm's physical structure, thereby establishing the MBEC, the lowest concentration capable of eradicating a pre-established biofilm (Masadeh et al. 2024; El-Telbany et al. 2022; Mohsenzadeh et al. 2025; Murugesan et al. 2018).

Other studies conducted both tests, demonstrating that PEG-coated ZnO-NP were effective in both preventing the onset of infection and eradicating mature biofilms (Thakral et al. 2023). Furthermore, some studies supplemented their results with Scanning Electron Microscopy (SEM) to observe massive structural damage to the extracellular matrix (Abdelghafar et al. 2022; El-Telbany et al. 2022); while others employed Hoechst staining for high-content screening (HCS) of live biofilms (Murugesan et al. 2018).

In general, studies incorporating biofilm assays have reported relevant results in both inhibiting initial formation and eradicating pre-established biofilm matrices when ZnO-NP were combined with antibiotics. Several hypotheses about the mechanisms of action can be correlated with these results: the ability of ZnO-NP to generate ROS and destabilize the extracellular matrix may contribute to better penetration of both ZnO-NP-antibiotic into the biofilm, potentially overcoming diffusion barriers that limit the effectiveness of antibiotics alone (Abdelghafar et al. 2022; Masoudi et al. 2024; Ibne Shoukani et al. 2024).

Furthermore, synergistic treatment can reduce the hydrophobicity index of the bacterial cell surface, compromising initial adhesion for biofilm formation. Repression of genes that regulate the biofilm was also observed, causing a dispersion of biofilm cells, leading to its disruption (Abdelghafar et al. 2022). Some studies also suggest that the methodology adopted can influence the delivery of the respective systems in biofilms. The results obtained in studies that used coatings with polyethylene glycol (Ibne Shoukani et al. 2024) or chitosan (Sathiyaseelan et al. 2023) demonstrated superior control in release kinetics, ensuring antibiofilm activity for prolonged periods.

Taken together, these findings indicate that while planktonic assays remain the primary screening strategy, the inclusion of biofilm models provides a more realistic assessment of antimicrobial performance. The results reported so far suggest that ZnO-NP-antibiotic systems may offer promising strategies for controlling biofilm-associated infections, although further studies are still required to evaluate safety, pharmacokinetics, and clinical applicability.

### Toxicological evaluation

The toxicological evidence identified across the included studies was limited and heterogeneous. In vitro cytotoxicity, in vivo evaluations, and safety-related outcomes were reported for only a subset of the investigated ZnO-NP-antibiotic formulations, whereas no clinical trial data were identified. The available evidence varied according to the formulation type and experimental approach, limiting direct comparisons of safety across ZnO-NP-antibiotic systems.

First, toxicity assessment was less frequently performed than antimicrobial evaluation. Regarding compounds in physical mixture, only 20% of them evaluated in vitro aspects, based on cytotoxic assays using vero cells or fibroblast cells (Masadeh et al. 2024; Khalil et al. 2025). Both researchers had discovered that the compounds exhibited low cellular toxicity, but the most significant finding was that cytotoxicity could be reduced when the compounds were combined with colistin (Masadeh et al. 2024).

Among studies evaluating physical mixture, only 20% incorporated in vivo tests assessments, both assays focused on the compounds' ability to treat infections: one involving the cornea (El-Telbany et al. 2022) and the other the skin (Abdelghafar et al. 2022). In these instances, in vivo toxic effects were not evaluated. Overall, the available data for this class of studies does not provide sufficient information to establish correlations regarding the safety of the developed compounds or to address clinical trials, which also remained without evaluation.

For conjugated compounds, only one of them underwent in vitro testing, specifically on fibroblasts (Mahgoub et al. 2025). The results were quite similar to those of (Masadeh et al. 2024), showing that the compound exhibited low toxicity to the cells; however, in the presence of the antibiotic, cell viability increased. This observation is relevant because it suggests that combination systems may not only enhance antimicrobial activity but also modulate the interaction of ZnO-NP with host cells. Several factors may contribute to this effect, including changes in nanoparticle surface chemistry, reduced direct contact between free ZnO-NP and mammalian cells, altered aggregation behavior, modified Zn^2^⁺ release, or changes in ROS generation after antibiotic association (Mahgoub et al. 2025; Bahadori Zade et al. 2024; Sathiyaseelan et al. 2023; Ibne Shoukani et al. 2024). However, for the conjugated compounds, no in vivo tests were conducted, nor other relevant aspects to conclude about their safety and clinical trials.

Regarding the encapsulated systems, cytotoxicity testing was performed on half of them. In the initial study, an in vitro test using the L-929 dermal cell line revealed that all tested hydrogels maintained cell viability above 80%, reaching values ​​exceeding 90% in some cases, thereby classifying them as non-cytotoxic (Bahadori Zade et al. 2024). Furthermore, blood compatibility was assessed via hemolysis test using rabbit blood to determine whether the developed compound would cause red blood cell rupture. The hemolysis index remained below 2% for all samples, indicating excellent hemocompatibility. An in vitro anti-inflammatory test was also conducted to evaluate the bio compound’s ability to inhibit bovine serum albumin denaturation, demonstrating that the developed compound exerted a significant protective effect (Bahadori Zade et al. 2024).

In another study, a cytotoxicity test was conducted using the baby hamster kidney (BHK21) cell line. The results showed that the developed system exhibited better cell viability than isolated ZnO-NP, with values ​​ranging from 90 to 95% even at the highest concentrations tested; this indicates that surface modification with PEG and the antibiotic reduced the inherent toxicity of the zinc oxide (Ibne Shoukani et al. 2024). A hemolysis test using human erythrocytes (Group O) was also performed to assess blood compatibility. While isolated pure ZnO-NP showed hemolysis rates between 5 and 7%, treatment with the developed system showed no significant hemolysis, rendering it biocompatible and safe for systemic circulation (Ibne Shoukani et al. 2024).

For the in vivo studies, a wound-healing test was conducted, during which the animals' body weight was regularly measured. The study reported no discernible changes in weight across any of the groups, suggesting an absence of acute systemic toxicity during the treatment period (Bahadori Zade et al. 2024). Subsequently, the inflammatory response was analyzed via RT-qPCR to measure the expression of pro-inflammatory genes such as IL-6 and IL-β1; it was observed that the group treated with the developed compound showed a significant reduction in the levels of these markers compared to the control, indicating an attenuation of inflammation at the wound site. In summary, this research employed a combination of cell viability tests, blood compatibility assays, and clinical monitoring of the animals to validate that the encapsulated system is biologically safe for topical application on wounds (Bahadori Zade et al. 2024).

In another study, in vivo tests were conducted to assess the compound's efficacy in healing wounds infected with *S. aureus*. Following the experiments, safety parameters were evaluated, such as histological analysis, to examine the morphological characteristics of the repaired skin and confirm the absence of adverse tissue damage resulting from contact with the developed compound (Ibne Shoukani et al. 2024). It was observed that combining the antibiotic with ZnO-NP improved the antibiotic's stability and enabled targeted delivery, thereby minimizing exposure of non-target tissues and ensuring a safer therapeutic profile. Furthermore, PEG coating reduced nanoparticle aggregation in the bloodstream, potentially lowering cellular toxicity and enhancing safety (Ibne Shoukani et al. 2024).

Regarding the results reported for the encapsulated systems, data concerning compound safety were available; however, but this potential advantage must be confirmed using standardized dose–response assays, multiple clinically relevant cell models, mechanistic toxicity endpoints, and other analysis referring directly to in vivo safety evaluation (Masadeh et al. 2024; El-Telbany et al. 2022; Khalil et al. 2025; Gómara and Ramón-García 2019).

Regarding the drug-loaded formulation, only in vivo tests were conducted. In this instance, safety and biological efficacy were evaluated using an excision wound model in albino rats; following treatment, hematoxylin and eosin staining was performed to observe the overall skin structure and identify potential acute histological alterations or damage to the epidermis and dermis (Saddik et al. 2022). Furthermore, the study employed a computerized image analysis system to measure epidermal thickness, ensuring that the treatment did not induce atrophy or disordered hyperproliferation. Thus, the safety of the developed compound was demonstrated by the absence of adverse tissue damage during the healing process in complex animal models, with safety validation based directly on the living organism's response and the histological integrity of the treated tissues (Saddik et al. 2022).

In general, i*n vivo* evaluations were less frequent and mainly focused on therapeutic outcomes rather than comprehensive toxicological profiling. For example, some studies investigated wound healing or infection control, reporting accelerated healing or improved response to infection treatment. These findings suggest potential therapeutic relevance, particularly for topical or localized applications. However, the available in vivo evidence remains limited and heterogeneous, and most studies did not provide detailed information on systemic toxicity, biodistribution, pharmacokinetics, organ accumulation, or long-term safety (Mahgoub et al. 2025; Sathiyaseelan et al. 2023; Masoudi et al. 2024).

This limitation is particularly relevant when considering the antimicrobial concentrations reported in vitro. Several studies reported effective ZnO-NP concentrations in the microgram-per-milliliter range; but it remains unclear whether these concentrations can be achieved and maintained at infection sites in vivo without inducing local or systemic toxicity. After administration, ZnO-NP may undergo aggregation, protein corona formation, partial dissolution with Zn^2^⁺ release, tissue retention, or clearance by mononuclear phagocyte systems, which can alter both antimicrobial activity and safety (Mahgoub et al. 2025; Sathiyaseelan et al. 2023; Ibne Shoukani et al. 2024).

The clinical significance of in vitro MIC reductions or synergistic interactions depends not only on microbiological potency, but also on whether effective concentrations are pharmacokinetically achievable under physiological conditions. This issue remains largely unresolved in the included studies, particularly because biodistribution, exposure duration, tissue accumulation, and elimination profiles were rarely assessed (Mahgoub et al. 2025; Gómara and Ramón-García 2019). The SYRCLE assessment (Hooijmans et al. 2014) further supports this limitation, showing that most in vivo studies had predominantly unclear risk of bias due to incomplete reporting of randomization, allocation concealment, random housing, and blinding procedures.

It is known that ZnO-NP induces the generation of ROS, oxidative stress, mitochondrial dysfunction, and potential inflammatory responses, effects that may be dose-dependent and strongly influenced by particle size, surface charge, aggregation state, and coating strategy (Albanese et al. 2012). Therefore, isolated cytotoxicity data, particularly when obtained under short-term exposure conditions, may underestimate the long-term biological impact. Furthermore, the limited number of in vivo investigations highlights a substantial translational bottleneck.

Systemic administration of metal oxide nanoparticles raises important questions about biodistribution, accumulation in organs (especially liver, spleen, and lungs), elimination pathways, and potential chronic toxicity. Without pharmacokinetic profiles and long-term exposure studies, it remains uncertain whether the concentration required to achieve antimicrobial synergy can be safely maintained in physiological settings (Albanese et al. 2012).

Another critical problem lies in the lack of standardized toxicity protocols across studies. Variability in nanoparticle synthesis methods, surface functionalization, dispersion media, dosage regimens, and biological models limit comparisons between studies and hinder reproducibility. This heterogeneity makes it difficult to establish safety thresholds or define structure–toxicity relationships that could guide the rational development of formulations (Pechyen et al. 2024).

## Limitations, evidence gaps, and future perspectives

Initially, the first limitation is, in fact, a potential limitation of the search strategy. Some eligible studies may not have been retrieved through the primary database searches because of the differences in indexing practices and the terminology used to describe ZnO-NP-antibiotic systems. This limitation was partially addressed by complementary manual searches of Google Scholar and reference lists, which allowed the identification of additional eligible studies. Nevertheless, the possibility that relevant studies may not still have been identified cannot be completely excluded.

The methodological heterogeneity of the included studies should be considered when interpreting the reported synergistic effects. Many investigations used a limited number of microbial strains, and some relied mainly on reference strains rather than resistant clinical isolates. In addition, synergy was not always assessed using standardized quantitative methods, such as checkerboard assays, FICI, or time-kill curves.

In many cases, synergistic activity was reported according to the interpretation adopted by the original authors, based on outcomes such as MIC/MBC reduction, increased inhibition zones, antibiofilm activity, enzyme inhibition, or statistical significance. Therefore, because this scoping review did not generate new experimental data, studies without FICI, time-kill assays, or equivalent quantitative interaction analyses were interpreted with caution as indicating enhanced antimicrobial activity, potentiation, or potential synergy, rather than definitive pharmacological synergy.

It is important to distinguish between mechanisms broadly described in the literature for ZnO-NP and mechanisms directly demonstrated in the specific combination studies included in this review. In many of the included articles, mechanisms such as ROS generation, Zn^2^⁺ release, membrane destabilization, efflux pump inhibition, and increased intracellular antibiotic accumulation were proposed or inferred from antimicrobial outcomes, rather than experimentally confirmed through dedicated mechanistic assays.

Although particle size was commonly described, the use and reporting of accepted physicochemical characterization techniques were inconsistent or absent in several studies. This is an important limitation because ZnO-NP size, morphology, crystallinity, surface chemistry, surface charge, aggregation state, synthesis method, dispersion conditions, antibiotic concentration, and microbiological protocols can directly influence antimicrobial performance and biological interactions. Therefore, incomplete characterization limits direct comparisons between studies and prevents the establishment of robust structure–activity relationships.

As the reported antimicrobial enhancement is promising, the current evidence should still be interpreted as predominantly exploratory and proof-of-concept. Future studies should adopt standardized antimicrobial interaction models, include resistant clinical isolates, provide complete physicochemical characterization of nanoparticles, and incorporate mechanistic and translational validation to better distinguish additive, potentiating, and truly synergistic effects.

Toxicological evidence also remains limited in depth and biological relevance. Most safety assessments were based on short-term cytotoxicity assays using a restricted number of cell models, such as fibroblasts, Vero cells, or baby hamster kidney cells. Even though these assays provide preliminary information on cell viability, they do not fully predict in vivo safety, tissue-specific toxicity, inflammatory responses, oxidative stress, genotoxicity, immune activation, biodistribution, or long-term effects. This limitation is particularly relevant because ZnO-NP may induce ROS generation, oxidative stress, mitochondrial dysfunction, inflammatory responses, and organ toxicity, and these effects may be dose-dependent and influenced by particle size, surface charge, aggregation state, Zn^2^⁺ release, dissolution kinetics, and coating strategy.

Similarly, the available in vivo studies were limited, heterogeneous, and focused primarily on therapeutic outcomes, such as infection control or wound healing, rather than comprehensive systemic toxicity. Systemic or repeated exposure to metal oxide nanoparticles raises important questions regarding biodistribution, accumulation in organs such as the liver, spleen, kidneys, and lungs, elimination pathways, nanoparticle dissolution, chronic toxicity, and long-term exposure risks. The lack of pharmacokinetic profiles, biodistribution analyses, organ accumulation studies, repeated-dose toxicity studies, hemocompatibility, immunogenicity, genotoxicity, and standardized toxicological endpoints represent a major translation gap.

Another important limitation is the lack of standardized toxicity protocols across studies. Variability in nanoparticle synthesis methods, surface functionalization, dispersion media, dosage regimens, exposure times, concentration units, biological models, and toxicity endpoints limits comparisons between studies and hinders reproducibility. This heterogeneity makes it difficult to establish safety thresholds or define structure–toxicity relationships that could guide the rational development of ZnO-NP-antibiotic formulations. Consequently, future studies should integrate antimicrobial efficacy with biologically relevant safety models and regulatory-oriented toxicity assessment.

The SYRCLE assessment indicated that the main limitation of the in vivo evidence was the predominance of unclear risk-of-bias judgments, mainly due to insufficient reporting of randomization, allocation concealment, random housing, and blinding procedures. These domains are not always routinely described in experimental antimicrobial studies, but they are essential for evaluating internal validity, reproducibility, and translational reliability. In this scenario, future in vivo investigations should report these methodological procedures more transparently, allowing a more robust assessment of bias and strengthening the preclinical evidence supporting ZnO-NP-antibiotic systems.

Beyond biological efficacy and toxicity, important pharmaceutical and regulatory barriers remain insufficiently addressed in the available literature. For ZnO-NP-antibiotic systems to advance toward clinical application, future studies must consider manufacturing reproducibility, batch-to-batch consistency, nanoparticle stability, scale-up feasibility, and quality control requirements. These aspects are particularly relevant because small variations in synthesis conditions, particle size distribution, aggregation state, surface charge, coating, drug loading, or release profile may substantially alter antimicrobial activity, toxicity, and biological performance.

In addition, the transition from laboratory-scale synthesis to GMP-compliant production represents a major translational challenge. Scalable manufacturing processes must ensure reproducible physicochemical properties, sterility, absence of contaminants, controlled endotoxin levels, long-term formulation stability, and predictable antibiotic release. Regulatory development would also require well-defined specifications for nanoparticle identity, purity, potency, safety, and performance. Consequently, although ZnO-NP-antibiotic systems show promising antimicrobial enhancement, their clinical translation will depend not only on microbiological efficacy, but also on robust pharmaceutical development, standardized quality control, and regulatory-oriented preclinical validation.

## Conclusion

Increasing antimicrobial resistance is progressively compromising the effectiveness of conventional antibiotic therapies, requiring innovative, yet safe and effective strategies for controlling bacterial infections. In this context, ZnO-NP have emerged as potential new antimicrobial agents; in addition to their intrinsic activity, they are also capable of enhancing the performance of antibiotics through various mechanisms such as cell membrane disruption, generation of reactive oxygen species, and modulation of bacterial metabolic pathways.

The studies analyzed in this review consistently demonstrated that the combination of ZnO-NP-antibiotic leads to a synergistic effect on antimicrobial activity, with a reduction in MIC and MBC values, an increase in inhibition zones, and a reduction and disruption of biofilms. Notably, synergistic effects were observed in multiple classes of antibiotics, including β-lactams, fluoroquinolones, polymyxins, and lincosamides. These findings suggest that the interaction between ZnO-NP-antibiotic is not restricted to a single antibiotic class or a specific type of microorganism. Synergistic effects have been reported in studies involving Gram-negative and Gram-positive bacteria.

From a formulation standpoint, most studies adopted simple co-administration approaches, followed by a few studies with functionalization between ZnO-NP and, finally, even fewer studies using polymers or hybrid materials in their formulation. This progression also reflects the evolution of methodologies over time. More recent research presents more innovative approaches such as the use of polymeric or hybrid materials.

However, regardless of the methodology employed, the studies still have little information regarding stability, controlled release, and definitive evidence demonstrating in vivo performance. This methodological imbalance represents a significant translational gap. Since many clinically relevant infections involve biofilm formation and complex host–pathogen interactions, future research must go beyond conventional susceptibility assays to establish the true therapeutic potential of these nanostructured combinations.

Thus, toxicological evaluation remains insufficiently explored. Although several studies have reported acceptable cytocompatibility profiles and, in some cases, reduced toxicity after antibiotic incorporation, standardized safety assessments and long-term studies are still needed before clinical translation can be considered.

Overall, ZnO-NP-antibiotic systems represent a strategy to enhance antibacterial therapy and potentially mitigate the development of resistance. However, advancement in this area will require more rigorous comparative studies, standardized biofilm models, comprehensive toxicological evaluation, and in vivo validation. Overcoming these gaps will be essential to translate promising in vitro synergistic effects into clinically viable and safe antimicrobial interventions.

## Supplementary Information

Supplementary Material 1.

## Data Availability

No data was used for the research described in the article.

## References

[CR1] Abdelghafar A, Yousef N, Askoura M. Zinc oxide nanoparticles reduce biofilm formation, synergize antibiotics action and attenuate *Staphylococcus aureus* virulence in host; an important message to clinicians. BMC Microbiol. 2022;22:244. 10.1186/s12866-022-02658-z. PMC9552502 36221053

[CR2] Albanese A, Tang PS, Chan WCW. The effect of nanoparticle size, shape, and surface chemistry on biological systems. Annu Rev Biomed Eng. 2012;14:1–16. 10.1146/annurev-bioeng-071811-150124. 22524388

[CR3] Anwar MA, Aqib AI, Ashfaq K, et al. Antimicrobial resistance modulation of MDR *E. coli* by antibiotic coated ZnO nanoparticles. Microb Pathog. 2020;148:104450. 10.1016/j.micpath.2020.104450. 32853679

[CR4] Azeem K, Fatima S, Ali A, et al. Biochemistry of bacterial biofilm: insights into antibiotic resistance mechanisms and therapeutic intervention. Life. 2025;15:1–23. 10.3390/life15010049. PMC11767195 39859989

[CR5] Bahadori Zade M, Abdollahi S, Raoufi Z, Zare Asl H. Synergistic antibacterial and wound healing effects of chitosan nanofibers with ZnO nanoparticles and dual antibiotics. Int J Pharm. 2024;666:124767. 10.1016/j.ijpharm.2024.124767. 39332456

[CR6] Cao L, Yan J, Luo T, et al. Antibacterial and fluorescent clear aligner attachment resin modified with chlorhexidine loaded mesoporous silica nanoparticles and zinc oxide quantum dots. J Mech Behav Biomed Mater. 2023;141:105817. 10.1016/j.jmbbm.2023.105817. 37015147

[CR7] Elekhnawy E, Almurshedi AS, Abdelkader DH, et al. Green synthesised zinc oxide nanoparticles reveal potent in vivo and in vitro antibacterial efficacy against *Proteus mirabilis* isolates. Int J Pharm. 2023;642:123111. 10.1016/j.ijpharm.2023.123111. 37302668

[CR8] El-Telbany M, Mohamed AA, Yahya G, et al. Combination of meropenem and zinc oxide nanoparticles; antimicrobial synergism, exaggerated antibiofilm activity, and efficient therapeutic strategy against bacterial keratitis. Antibiotics (Basel). 2022;11:1374. 10.3390/antibiotics11101374. PMC9598448 36290032

[CR9] Fadwa AO, Alkoblan DK, Mateen A, Albarag AM. Synergistic effects of zinc oxide nanoparticles and various antibiotics combination against *Pseudomonas aeruginosa* clinically isolated bacterial strains. Saudi J Biol Sci. 2021;28:928–35. 10.1016/j.sjbs.2020.09.064. PMC7785452 33424384

[CR10] Fathi Azar Khavarani M, Najafi M, Shakibapour Z, Zaeifi D. Kinetics activity of *Yersinia intermedia* against ZnO nanoparticles either synergism antibiotics by double-disc synergy test method. Iran J Biotechnol. 2016;14:40–4. 10.15171/ijb.1184. PMC5435012 28959316

[CR11] Gelen-Gungor D, Nigiz Ş, Özkul C, et al. Co-delivery of azithromycin and nisin through liposomes for skin infection to reduce antimicrobial drug resistance. Int J Pharm. 2025;679:125764. 10.1016/j.ijpharm.2025.125764. 40414326

[CR12] Ghasemi F, Jalal R. Antimicrobial action of zinc oxide nanoparticles in combination with ciprofloxacin and ceftazidime against multidrug-resistant *Acinetobacter baumannii*. J Glob Antimicrob Resist. 2016;6:118–22. 10.1016/j.jgar.2016.04.007. 27530853

[CR13] Gómara M, Ramón-García S. The FICI paradigm: correcting flaws in antimicrobial in vitro synergy screens at their inception. Biochem Pharmacol. 2019;163:299–307. 10.1016/j.bcp.2019.03.001 30836058

[CR14] Hasannasab M, Nourmohammadi J, Dehghan MM, Ghaee A. Immobilization of bromelain and ZnO nanoparticles on silk fibroin nanofibers as an antibacterial and anti-inflammatory burn dressing. Int J Pharm. 2021;610:121227. 10.1016/j.ijpharm.2021.121227. 34699950

[CR15] Hayles A, Nguyen HN, Alemie M, et al. Electrostatic charge at the biomaterial-pathogen interface influences antibiotic efficacy. Adv Biotechnol. 2025;3:10. 10.1007/s44307-025-00061-z. PMC11965051 40175809

[CR16] Hooijmans CR, Rovers MM, de Vries RB, et al. SYRCLE’s risk of bias tool for animal studies. BMC Med Res Methodol. 2014;14:43. 10.1186/1471-2288-14-43. PMC4230647 24667063

[CR17] Ibne Shoukani H, Nisa S, Bibi Y, et al. Ciprofloxacin loaded PEG coated ZnO nanoparticles with enhanced antibacterial and wound healing effects. Sci Rep. 2024;14:4689. 10.1038/s41598-024-55306-z. PMC11322433 38409460

[CR18] Iheme CI, Elemike EE, Igwe CU, et al. Synthesis, characterization, radical scavenging properties of zinc oxide nanoparticles and inhibitory effect of ZnONPs-ciprofloxacin nanoconjugates on multidrug-resistant Staphylococcus aureus (MRSA) enzyme. Inorg Chem Commun. 2024;160. 10.1016/j.inoche.2023.111864.

[CR19] Khalil MA, Alzaidi TM, Alsharbaty MHM, et al. Synergistic antibacterial and antibiofilm effects of clindamycin and zinc oxide nanoparticles against pathogenic oral *Bacillus* species. Pathogens. 2025;14:138. 10.3390/pathogens14020138. PMC11858533 40005514

[CR20] Khuzaimah I, Santosa SJ, Pambudi FI, et al. Physicochemical and antibacterial properties of ZnO nanoparticles-modified mineral trioxide aggregate hydrated with antibiotic/chitosan solution. Braz Dent Sci. 2025;28:e4595. 10.4322/bds.2025.e4595.

[CR21] Li Y, Zhen J, Tian Q, et al. One step synthesis of positively charged gold nanoclusters as effective antimicrobial nanoagents against multidrug-resistant bacteria and biofilms. J Colloid Interface Sci. 2020;569:235–43. 10.1016/j.jcis.2020.02.084. 32113021

[CR22] Li J, Jia T, Yang L. Targeting anti-virulence factor strategies of bacterial pathogens. Biosaf Health. 2025;7:1–4. 10.1016/j.bsheal.2025.01.006. PMC12861749 41635302

[CR23] Mahgoub SM, Mohamed EA, Aziz SAAA, et al. Synergistic potential of clindamycin hydrochloride loaded on zinc oxide nanoparticles: a novel approach to combat multidrug-resistant infections. Sci Rep. 2025;15:44665. 10.1038/s41598-025-30573-6. PMC12749461 41455716

[CR24] Marchianò V, Matos M, Serrano-Pertierra E, et al. Vesicles as antibiotic carrier: state of art. Int J Pharm. 2020;585:119478. 10.1016/j.ijpharm.2020.119478. 32473370

[CR25] Masadeh MM, Bany-Ali NM, Khanfar MS, et al. Synergistic antibacterial effect of ZnO nanoparticles and antibiotics against multidrug-resistant biofilm bacteria. Curr Drug Deliv. 2024;22:92–106. 10.2174/0115672018279213240110045557. 38231065

[CR26] Masoudi M, Mashreghi M, Zenhari A, Mashreghi A. Combinational antimicrobial activity of biogenic TiO2 NP/ZnO NPs nanoantibiotics and amoxicillin-clavulanic acid against MDR-pathogens. Int J Pharm. 2024;652:123821. 10.1016/j.ijpharm.2024.123821. 38242259

[CR27] Mehta M, Allen-Gipson D, Mohapatra S, et al. Study on the therapeutic index and synergistic effect of Chitosan-zinc oxide nanomicellar composites for drug-resistant bacterial biofilm inhibition. Int J Pharm. 2019;565:472–80. 10.1016/j.ijpharm.2019.05.003. 31071421

[CR28] Mohsenzadeh A, Mohsenzadeh H, Mohammadi A, et al. Synergistic effects of zinc oxide nanoparticles and meropenem on biofilm formation in *Pseudomonas aeruginosa*. Cell Mol Biomed Rep. 2025;5:121–34. 10.55705/cmbr.2025.481668.1280.

[CR29] Murugesan G, Latha N, Suganya K, et al. Stimulus-responsive zinc oxide-functionalized macromolecular humic acid nanocarrier for enhancement of antibacterial activity of ciprofloxacin hydrochloride. Int J Biol Macromol. 2018;114:1109–16. 10.1016/j.ijbiomac.2018.03.120. 29578024

[CR30] Ouzzani M, Hammady H, Fedorowicz Z, Elmagarmid A. Rayyan—a web and mobile app for systematic reviews. Syst Rev. 2016;5:210. 10.1186/s13643-016-0384-4. PMC5139140 27919275

[CR31] Pasquet J, Chevalier Y, Couval E, et al. Antimicrobial activity of zinc oxide particles on five micro-organisms of the challenge tests related to their physicochemical properties. Int J Pharm. 2014;460:92–100. 10.1016/j.ijpharm.2013.10.031. 24211859

[CR32] Pastore V, Frison J, Pesce C, et al. Development of nanovehicles for co-delivery of colistin and ArnT inhibitors. Int J Pharm. 2025;675:125515. 10.1016/j.ijpharm.2025.125515. 40147699

[CR33] Pechyen C, Tangnorawich B, Toommee S, et al. Green synthesis of metal nanoparticles, characterization, and biosensing applications. Sens Int. 2024;5:100287. 10.1016/j.sintl.2024.100287.

[CR34] Peters MDJ, Godfrey CM, Khalil H, et al. Guidance for conducting systematic scoping reviews. Int J Evid Based Healthc. 2015;13:141–6. 10.1097/XEB.0000000000000050. 26134548

[CR35] Ravi J, Gunasekar B, Basha J. Synergistic antimicrobial activity of colistin and amikacin with zinc oxide nanoparticles and their posttranscriptional regulation of *mcr-1* gene expression in colistin-resistant *Klebsiella pneumoniae*. Microb Drug Resist. 2025;31:201–10. 10.1089/mdr.2024.0117. 40488625

[CR36] Saddik MS, Elsayed MMA, El-Mokhtar MA, et al. Tailoring of novel azithromycin-loaded zinc oxide nanoparticles for wound healing. Pharmaceutics. 2022;14:111. 10.3390/pharmaceutics14010111. PMC8780377 35057019

[CR37] Saikia S, Chetia P. Antibiotics: from mechanism of action to resistance and beyond. Indian J Microbiol. 2024;64:821–45. 10.1007/s12088-024-01285-8. PMC11399512 39282166

[CR38] Sathiyaseelan A, Saravanakumar K, Zhang X, et al. Ampicillin-resistant bacterial pathogens targeted chitosan nano-drug delivery system (CS-AMP-P-ZnO) for combinational antibacterial treatment. Int J Biol Macromol. 2023;237:124129. 10.1016/j.ijbiomac.2023.124129. 36958450

[CR39] Seifi S, Shahverdi M, Shaygani H, et al. Fabrication of gelatin-based antibacterial bilayer wound dressing using direct writing and electrospinning methods. Int J Pharm. 2024;659:124274. 10.1016/j.ijpharm.2024.124274. 38802029

[CR40] Sharif M, Tunio SA, Bano S. Synergistic effects of zinc oxide nanoparticles and conventional antibiotics against methicillin resistant *Staphylococcus aureus*. Adv Life Sci. 2021;8:167–71. 10.62940/als.v8i2.1092.

[CR41] Sharma N, Jandaik S, Kumar S. Synergistic activity of doped zinc oxide nanoparticles with antibiotics: ciprofloxacin, ampicillin, fluconazole and amphotericin B against pathogenic microorganisms. An Acad Bras Cienc. 2016;88:1689–98. 10.1590/0001-3765201620150713. 27737336

[CR42] Thakral F, Singh Tuli H, Gupta S, et al. Synergistic anti-bacterial effects of green synthesized zinc oxide nanoparticles with levofloxacin. J King Saud Univ Sci. 2023;35:102905. 10.1016/j.jksus.2023.102905.

[CR43] Thamer R, Alsammak E. Synergistic effect of zinc oxide nanoparticles and vancomycin on methicillin resistant *Staphylococcus aureus*. J Life Bio Sci Res. 2021;2:01–6. 10.38094/jlbsr1332.

[CR44] Tricco AC, Lillie E, Zarin W, et al. PRISMA extension for scoping reviews (PRISMA-ScR): checklist and explanation. Ann Intern Med. 2018;169:467–73. 10.7326/M18-0850. 30178033

[CR45] Vasile BS, Oprea O, Voicu G, et al. Synthesis and characterization of a novel controlled release zinc oxide/gentamicin–chitosan composite with potential applications in wounds care. Int J Pharm. 2014;463:161–9. 10.1016/j.ijpharm.2013.11.035. 24291078

[CR46] Ybarra DE, Quezada C, Guarín YA, et al. Self-assembled PAMAM-G4 dendrimer nanoparticles with Phloxine B as photosensitizer for antimicrobial photodynamic therapy. Int J Pharm. 2025;675:125534. 10.1016/j.ijpharm.2025.125534. 40164413

[CR47] Yu Y, Alkasir R, Farrukh A, et al. Synergistic antibacterial potential of Zno-Nps with different antibiotics against multidrug-resistant *Escherichia coli* and *Pseudomonas aeruginosa*. Pol J Environ Stud. 2024;33:4893–902. 10.15244/pjoes/176158.

