# Supplementary material for: Synergistic effect of zinc oxide nanoparticles and antibiotics: a strategy to combat bacterial resistance
Source: Adv Biotechnol (Singap). 2026 Oct 26;4(4):44. doi: 10.1007/s44307-026-00137-4 (PMC13710522; doi:10.1007/s44307-026-00137-4)
Supplement: Supplementary file 1 [file 44307_2026_137_MOESM1_ESM.docx]

**Table S1.** Search strategies and results obtained from each database.

The search strategies are presented exactly as executed in each database. No publication date, language, or other database filters were applied.

| **Database** | **Search strategy** | **Results** |
| --- | --- | --- |
| **PubMed** | **Query 1:** ((((((((((("Anti-Bacterial Agents"[MeSH Terms]) OR ("Anti-bacterial Agents"[Title/Abstract])) OR ("Silver sulfadiazine"[Title/Abstract])) OR ("Sulfadiazine"[Title/Abstract])) OR ("Sulfapyridine"[Title/Abstract])) OR ("Sulfa drugs"[Title/Abstract])) OR ("Sulfonamides"[Title/Abstract])) OR ("Drug Resistance, Bacterial"[Title/Abstract])) OR ("Drug Resistance, Bacterial"[MeSH Terms])) OR ("Tetracyclines"[Title/Abstract])) OR ("Aminoglycosides"[Title/Abstract])) OR ("Penicillin"[Title/Abstract])  **Query 2:** ((("Zinc oxide nanoparticles"[Title/Abstract]) OR ("ZnO nanoparticles"[Title/Abstract])) OR ("ZnO NPs"[Title/Abstract])) OR ("ZnO NP"[Title/Abstract])  **Query 3:** (#1) AND (#2) | 1,819 |
| **Scopus** | TITLE-ABS-KEY ( "Anti-Bacterial Agents" OR "Silver sulfadiazine" OR "Sulfadiazine" OR "Sulfapyridine" OR "Sulfa drugs" OR "Sulfonamides" OR "Drug Resistance, Bacterial" OR "Tetracyclines" OR "Aminoglycosides" OR "Penicillin" ) AND TITLE-ABS-KEY ( "Zinc oxide nanoparticles" OR "ZnO nanoparticles" OR "ZnO NPs" OR "ZnO NP" ) | 1,694 |
| **Web of Science** | **Query 1:** TS=("Anti-Bacterial Agents" OR "Anti-bacterial Agents" OR "Silver sulfadiazine" OR "Sulfadiazine" OR "Sulfapyridine" OR "Sulfa drugs" OR "Sulfonamides" OR "Drug Resistance, Bacterial" OR "Tetracyclines" OR "Aminoglycosides" OR "Penicillin")  **Query 2:** TS=("Zinc oxide nanoparticles" OR "ZnO nanoparticles" OR "ZnO NPs" OR "ZnO NP")  **Query 3:** #1 AND #2 | 1,540 |

**Table S2.** Studies excluded after full-text screening and reasons for exclusion.

| **Title** | **Authors** | **DOI** | **Reasons for exclusion** |
| --- | --- | --- | --- |
| Biosynthesized ZnO NPs loaded-electrospun PVA/sodium alginate/glycine nanofibers: synthesis, spinning optimization and antimicrobial activity evaluation | Kenawy *et al.*, 2025 | 10.1038/s41598-024-81939-1 | Synergistic effects with other materials |
| Mechanochemically reprogrammed porous tantalum with synergistic antibacterial and osteogenic properties for the treatment of infectious bone defects | Sun *et al.*, 2025 | 10.1186/s12951-025-03610-9 | Synergistic effects with other materials |
| ZnO-Cu/Mn nanozyme for rescuing the intestinal homeostasis in *Salmonella*-induced colitis | Wu *et al.*, 2025 | 10.1186/s12951-025-03283-4 | Synergistic effects with metals |
| Biological performance of ZnO-doped hydroxyapatite coatings on PEEK: *in vitro* antibacterial, cytotoxic, and osteogenic assessment for orthopedic implants | Xu *et al*., 2025 | 10.1088/1748-605X/ae0ee9 | Synergistic effects with other materials |
| Curcumin-loaded polyvinyl alcohol/nanocellulose/ nano zinc oxide nanofiber membranes with enhanced strength, moisture resistance, and antibacterial properties | Ji *et al.*, 2025 | 10.1016/j.ijbiomac.2025.145989 | Synergistic effects with other materials |
| Sustainable synthesis of gold-embedded ZnO nanoparticles from natural extract for environmental and biomedical applications | Hussein *et al.*, 2025 | 10.1007/s13399-025-06825-w | Study of antibiotic removal in nature |
| Near-infrared light triggered bio-inspired enhanced natural silk fibroin nanofiber composite scaffold for photothermal therapy of periodontitis | Zheng *et al.,* 2025 | 10.1016/j.colsurfb.2025.114607 | Synergistic effects with other materials |
| Core-Shell ZnO2@Cerium-Based Metal-Organic Framework with Low Turnover, Dual-Catalytic Activity for Biosafe Biofilm Dispersal and Immune Modulation | Wu *et al.*, 2025 | 10.1021/acsami.5c08974 | Synergistic effects with metals |
| Synthesis and characterization of bamboo stem porous activated carbon/chitosan/zinc oxide (BSPAC/CS/ZnO) ternary nanocomposite for enhancement of biological activities | Cinnathambi Subramani *et al.*, 2025 | 10.1016/j.ijbiomac.2025.141041 | Synergistic effects with other materials |
| Bioconjugated zinc oxide–quercetin nanocomposite enhances the selectivity and anti-biofilm activity of ZnO nanoparticles against *Staphylococcus* species | Choi *et al.,* 2025 | 10.1007/s12257-024-00136-z | Synergistic effects with other materials |
| Bacteria-Inspired Synthesis of Silver-Doped Zinc Oxide Nanocomposites: A Novel Synergistic Approach in Controlling Biofilm and Quorum-Sensing-Regulated Virulence Factors in *Pseudomonas aeruginosa* | Karthikeyan *et al.*, 2025 | 10.3390/antibiotics14010059 | Synergistic effects with metals |
| Plasmon-Enhanced Photocatalysis and Antimicrobial Activity of Green-Synthesized Ag-Decorated ZnO Nanoparticles Using *Mentha pulegium* Extract | Al Aaraji *et al.*, 2025 | 10.1007/s11468-025-03000-9 | Synergistic effects with metals |
| Synergistic effects of modified zinc oxide nanoparticle in a hybrid chitosan-gelatin hydrogel for bone regeneration. | Hia *et al.,* 2025 | 10.1016/j.ijbiomac.2025.144490 | Synergistic effects with other materials |
| Cardamom essential oil-loaded zinc oxide nanoparticles: A sustainable antimicrobial strategy against multidrug-resistant foodborne pathogens. | Sobhy *et al.,* 2025 | 10.1016/j.micpath.2025.107661 | Synergistic effects with other materials |
| Efficiency of CaZn₂(OH)₆·2H₂O and ZnO nanoparticles in photocatalytic degradation of amoxicillin after multiple cycles. | Lázaro-Mass *et al.,* 2025 | 10.1016/j.chemosphere.2024.143577 | Study of antibiotic removal in nature |
| Green Synthesis of Silver-Doped ZnO Nanoparticles from *Adiantum venustum* D. Don (*Pteridaceae*): Antimicrobial and Antioxidant Evaluation. | Noor *et al.,* 2025 | 10.1002/jobm.202400543 | Synergistic effects with metals |
| Synergistic effect of zinc oxide-cinnamic acid nanoparticles for wound healing management: *in vitro* and zebrafish model studies. | Tayyeb *et al.*, 2024 | 10.1186/s12896-024-00906-w | Synergistic effects with other materials |
| One-step spray pyrolysis synthesis of ZnO/Ag hollow spheres for enhanced visible-light-driven antibacterial applications and wound healing | Miao *et al.*, 2024 | 10.1039/d4dt02581d | Synergistic effects with metals |
| Antimicrobial Activity of ZnS and ZnO-TOP Nanoparticles Against Pathogenic Bacteria | Bouasla *et al.*, 2024 | 10.1002/cbdv.202400724 | Synergistic effects with other materials |
| Green synthesis of Characterized Bio-functionalized ZnO Nanoparticles from *Terminalia catappa* (Almond) Methanol Leaf Extract and their Potential Antioxidant and Antibacterial Properties | Momoh *et al.,* 2024 | 10.26538/tjnpr/v8i11.45 | Zinc oxide nanoparticles only |
| Phytofabrication of biocompatible chitosan-based ZnO nanocomposite aided by *Cissus quadrangularis* extract enriched with antimicrobial and antioxidant potential | Umaiya Bharathi *et al.,* 2024 | 10.1016/j.ijbiomac.2024.132677 | Synergistic effects with other materials |
| Optical tweezers for probing the interactions of ZnO and Ag nanoparticles with *E. coli* | Hinge *et al.*, 2024 | 10.1007/s00203-024-03964-y | Synergistic effects with metals |
| Photocatalytic and antibacterial activity of green synthesized and immobilized zinc oxide nanoparticles for the removal of sulfadiazine and acetaminophen: Effect of operational parameters and degradation pathway | Sanjeev *et al.*, 2024 | 10.1016/j.jece.2024.112649 | Study of antibiotic removal in nature |
| Alkaline protease based hydrothermal synthesis of novel Pd/CuO/ZnO nanocomposite: A new entry into photocatalytic and biomedical applications | Qurashi *et al.*, 2024 | 10.1016/j.ijbiomac.2024.131155 | Synergistic effects with metals |
| Diffusion-driven fabrication of calcium and phosphorous-doped zinc oxide heterostructures on titanium to achieve dual functions of osteogenesis and prevent bacterial infections | Ullah *et al.*, 2024 | 10.1016/j.actbio.2023.12.046 | Synergistic effects with other materials |
| Chitosan-*Aloe Vera* Composition Loaded with Zinc Oxide Nanoparticles for Wound Healing: *In Vitro* and *In Vivo* Evaluations. | Alawadi *et al.,* 2024 | 10.1049/2024/6024411 | Synergistic effects with other materials |
| Central composite design and mechanism of antibiotic ciprofloxacin photodegradation under visible light by green hydrothermal synthesized cobalt-doped zinc oxide nanoparticles. | Hassaan *et al.*, 2024 | 10.1038/s41598-024-58961-4 | Study of antibiotic removal in nature |
| Green synthesis of zinc oxide nanoparticles (ZnO-NPs) by *Streptomyces baarnensis* and its active metabolite (Ka): a promising combination against multidrug-resistant ESKAPE pathogens and cytotoxicity. | Kalaba *et al.,* 2024 | 10.1186/s12866-024-03392-4 | Zinc oxide nanoparticles only |
| Antibacterial and antioxidative hydrogel dressings based on tannic acid-gelatin/oxidized sodium alginate loaded with zinc oxide nanoparticles for promoting wound healing. | Liu *et al*., 2024 | 10.1016/j.ijbiomac.2024.135177 | Synergistic effects with other materials |
| Antimicrobial GL13K Peptide-Decorated ZnO Nanoparticles to Treat Bacterial Infections. | Zhang *et al.*, 2024 | 10.1021/acs.langmuir.4c03206 | Synergistic effects with other materials |
| Rutin-coated zinc oxide nanoparticles: a promising antivirulence formulation against pathogenic bacteria. | Alidoust *et al.,* 2024 | 10.1007/s11274-024-03984-2 | Synergistic effects with other materials |
| Cytotoxicity and Antimicrobial Efficacy of Fe-, Co-, and Mn-Doped ZnO Nanoparticles. | Yin *et al.*, 2024 | 10.3390/molecules29245966 | Synergistic effects with metals |
| Therapeutic effect of ZnO NPs–polyhexanide-hydrogel on *Staphylococcus aureus*-induced skin wound infection in mice | Zhu *et al.*, 2024 | 10.1080/09205063.2024.2340819 | Synergistic effects with other materials |
| Study of Cytotoxic and Antibacterial Activity of Ag- and Mg-Dual-Doped ZnO Nanoparticles. | Azizi et al., 2024 | 10.1002/open.202300093 | Synergistic effects with metals |
| Fabrication of Cu/ZnO-loaded chitosan hydrogel for an effective wound dressing material to advance wound care and healing efficiency after caesarean section surgery | Wang *et al.*, 2024 | 10.1111/iwj.14366 | Synergistic effects with metals |
| Introducing the antibacterial and photocatalytic degradation potentials of biosynthesized chitosan, chitosan-ZnO, and chitosan-ZnO/PVP nanoparticles. | Aouadi *et al.*, 2024 | 10.1038/s41598-024-65579-z | Synergistic effects with other materials |
| Comparative study of zinc oxide nanoparticles synthesized through biogenic and chemical routes with reference to antibacterial, antibiofilm and anticancer activities. | Prakashkumar et al., 2023 | 10.1016/j.envres.2022.115136 | Zinc oxide nanoparticles only |
| *Ferula latisecta* gels for synthesis of zinc/silver binary nanoparticles: antibacterial effects against gram-negative and gram-positive bacteria and physicochemical characteristics. | Es-Haghi *et al.*, 2024 | 10.1186/s12896-024-00878-x | Synergistic effects with metals |
| Enhanced wound healing properties of biodegradable PCL/alginate core-shell nanofibers containing *Salvia abrotanoides* essential oil and ZnO nanoparticles. | Sasan *et al.,* 2024 | 10.1016/j.ijbiomac.2024.135152 | Synergistic effects with other materials |
| AMP-Coated TiO2 Doped ZnO Nanomaterials Enhanced Antimicrobial Activity and Efficacy in Otitis Media Treatment by Elevating Hydroxyl Radical Levels | Bai *et al.,* 2024 | 10.2147/IJN.S449888 | Synergistic effects with other materials |
| Antibacterial and fluorescent clear aligner attachment resin modified with chlorhexidine loaded mesoporous silica nanoparticles and zinc oxide quantum dots | Cao *et al.,* 2023 | 10.1016/j.jmbbm.2023.105817 | Synergistic effects with other materials |
| Improved Antibacterial Properties of Polylactic Acid-Based Nanofibers Loaded with ZnO-Ag Nanoparticles through Pore Engineering | Su *et al.,* 2023 | 10.1021/acsami.3c06791 | Synergistic effects with metals |
| Development of ZnO/selenium nanoparticles embedded chitosan-based anti-bacterial wound dressing for potential healing ability and nursing care after paediatric fracture surgery | Ruan *et al.,* 2023 | 10.1111/iwj.13947 | Synergistic effects with other materials |
| Efficient clearance of periodontitis pathogens by *S. gordonii* membrane-coated H2O2 self-supplied nanocomposites in a “Jenga” style | Cao *et al.,* 2023 | 10.1039/d3bm00641g | Synergistic effects with other materials |
| Hybrid nanomaterial composed of chitosan, curcumin, ZnO and TiO2 for antibacterial therapies | Karthikeyan *et al.,* 2023 | 10.1016/j.ijbiomac.2023.124814 | Synergistic effects with other materials |
| Antimicrobial Activity of Biogenic Metal Oxide Nanoparticles and Their Synergistic Effect on Clinical Pathogens | Francis *et al.,* 2023 | 10.3390/ijms24129998 | Synergistic effects with metals |
| Bifunctional electrospun poly (L-lactic acid) membranes incorporating black phosphorus nanosheets and nano-zinc oxide for enhanced biocompatibility and antibacterial properties in catheter materials | Xu *et al.,* 2023 | 10.1016/j.jmbbm.2023.105884 | Synergistic effects with other materials |
| Synergistic Effects of Silicate-Platelet Supporting Ag and ZnO, Offering High Antibacterial Activity and Low Cytotoxicity. | Chang *et al.,* 2023 | 10.3390/ijms24087024 | Synergistic effects with metals |
| Eco synthesized chitosan/zinc oxide nanocomposites as the next generation of nano-delivery for antibacterial, antioxidant, antidiabetic potential, and chronic wound repair. | Halarnekar *et al.,* 2023 | 10.1016/j.ijbiomac.2023.124764 | Synergistic effects with other materials |
| Biofabrication of Mg-doped ZnO nanostructures for hemolysis and antibacterial properties. | Al-Odayni *et al.,* 2023 | 10.1007/s00449-023-02937-7 | Synergistic effects with metals |
| Boosting photo-induced antimicrobial activity of lignin nanoparticles with curcumin and zinc oxide | Perveen *et al.,* 2023 | 10.1016/j.ijbiomac.2023.127433 | Synergistic effects with other materials |
| Green synthesis of Cu-Mn co-incorporated ZnO nanoparticles for antibacterial and photocatalytic applications. | Butt *et al.,* 2023 | 10.1002/jemt.24386 | Synergistic effects with metals |
| Combination of 2-tert-Butyl-1,4-Benzoquinone (TBQ) and ZnO Nanoparticles, a New Strategy to Inhibit Biofilm Formation and Virulence Factors of *Chromobacterium violaceum* | Liu *et al.,* 2023 | 10.1128/msphere.00597-22 | Synergistic effects with other materials |
| Optical and biological properties of MgO/ZnO nanocomposite derived via eggshell membrane: a bio-waste approach | Suvaitha *et al.,* 2023 | 10.1007/s00449-022-02811-y | Synergistic effects with metals |
| Biosynthesis, characterization and antimicrobial activity of zinc oxide and nickel doped zinc oxide nanoparticles using *Euphorbia abyssinica* bark extract. | Faye *et al.,* 2022 | 10.1049/nbt2.12072 | Synergistic effects with metals |
| What happens when nanoparticles encounter bacterial antibiotic resistance? | Xu *et al.,* 2023 | 10.1016/j.scitotenv.2023.162856 | Literature review |
| Plant-mediated synthesis of silver-doped zinc oxide nanoparticles and evaluation of their antimicrobial activity against bacteria cause tooth decay. | Mortezagholi *et al.,* 2022 | 10.1002/jemt.24207 | Synergistic effects with metals |
| Synergistic Effects of Selenium and Zinc Oxide Nanoparticles on Growth Performance, Hemato-biochemical Profile, Immune and Oxidative Stress Responses, and Intestinal Morphometry of Nile Tilapia (*Oreochromis niloticus*). | Ghazi *et al.,* 2022 | 10.1007/s12011-021-02631-3 | Synergistic effects with metals |
| A promising antimicrobial bio nanocomposite-based poly(3-hydroxybutyrate-co-3-hydroxyvalerate) reinforced silver doped zinc oxide nanoparticles | Ibrahim *et al.,* 2022 | 10.1038/s41598-022-17470-y | Synergistic effects with metals |
| Synergistic effect of ZnO nanoparticles and hesperidin on the antibacterial properties of chitosan | Erol *et al.,* 2022 | 10.1080/09205063.2022.2099668 | Synergistic effects with other materials |
| Antibacterial Activity Evaluation of ZnO, CuO, and TiO2 Nanoparticles in Solution and Thin Films | Mielcarek *et al.,* 2022 | 10.1007/978-1-0716-2473-9_4 | Synergistic effects with metals |
| Enhanced Activity and Sustained Release of Protocatechuic Acid, a Natural Antibacterial Agent, from Hybrid Nanoformulations with Zinc Oxide Nanoparticles. | AbouAitah *et al.,* 2021 | 10.3390/ijms22105287 | Synergistic effects with other materials |
| Synthesis, characterization, and evaluation of antibacterial efficacy of rhamnolipid-coated zinc oxide nanoparticles against *Staphylococcus aureus*. | Malakar *et al.*, 2021 | 10.1007/s11274-021-03160-w | Synergistic effects with other materials |
| Exploration of the antibacterial and wound healing potential of a PLGA/silk fibroin based electrospun membrane loaded with zinc oxide nanoparticles. | Khan *et al.,* 2021 | 10.1039/d0tb02822c | Synergistic effects with other materials |
| Synergetic effect of Sn doped ZnO nanoparticles synthesized via ultrasonication technique and its photocatalytic and antibacterial activity. | Selvinsimpson *et al.,* 2021 | 10.1016/j.envres.2021.111115 | Synergistic effects with metals |
| Synergistic biocidal effects of metal oxide nanoparticles-assisted ultrasound irradiation: Antimicrobial sonodynamic therapy against *Streptococcus mutans* biofilms | Pourhajibagher *et al.,* 2021 | 10.1016/j.pdpdt.2021.102432 | Synergistic effects with metals |
| High synergistic antibacterial, antibiofilm, antidiabetic and antimetabolic activity of *Withania somnifera* leaf extract-assisted zinc oxide nanoparticle. | Malaikozhundan *et al.,* 2020 | 10.1007/s00449-020-02346-0 | Zinc oxide nanoparticles only |
| Preparation and antimicrobial activity of ZnO-NPs coated cotton/starch and their functionalized ZnO-Ag/cotton and Zn(II) curcumin/cotton materials. | El-Nahhal *et al.,* 2020 | 10.1038/s41598-020-61306-6 | Synergistic effects with metals |
| Preparation, characterization and antibacterial mechanism of the chitosan coatings modified by Ag/ZnOmicrospheres | Wei *et al.,* 2020 | 10.1002/jsfa.10605 | Synergistic effects with metals |
| Hybrid chitosan-ZnO nanoparticles coated with a sonochemical technique on silk fibroin-PVA composite film: A synergistic antibacterial activity. | Patil *et al.,* 2019 | 10.1016/j.ijbiomac.2018.09.090 | Synergistic effects with other materials |
| Study on the therapeutic index and synergistic effect of Chitosan-zinc oxide nanomicellar composites for drug-resistant bacterial biofilm inhibition | Mehta *et al.*, 2019 | 10.1016/j.ijpharm.2019.05.003 | Synergistic effects with other materials |
| Biopolymer gelatin-coated zinc oxide nanoparticles showed high antibacterial, antibiofilm and anti-angiogenic activity. | Divya *et al.,* 2018 | 10.1016/j.jphotobiol.2017.11.008 | Synergistic effects with other materials |
| Nanocomposite films based on CMC, okra mucilage and ZnO nanoparticles: Physico mechanical and antibacterial properties. | Mohammadi *et al.,* 2018 | 10.1016/j.carbpol.2017.10.045 | Synergistic effects with other materials |
| Bactericidal impact of Ag, ZnO and mixed AgZnO colloidal nanoparticles on H(37)Rv *Mycobacterium tuberculosis* phagocytized by THP-1 cell lines. | Jafari *et al.,* 2017 | 10.1016/j.micpath.2017.07.010 | Synergistic effects with metals |
| Disinfection of Multidrug Resistant *Escherichia coli* by Solar-Photocatalysis using Fe-doped ZnO Nanoparticles. | Das *et al*., 2017 | 10.1038/s41598-017-00173-0 | Synergistic effects with metals |
| Mechanical and antibacterial properties of novel high-performance chitosan/nanocomposite films. | Youssef *et al.*, 2015 | 10.1016/j.ijbiomac.2015.02.016 | Synergistic effects with other materials |
| Synergistic antibacterial activity of nanohybrid materials ZnO-Ag and ZnO-Au: Synthesis, characterization, and comparative analysis of undoped and doped ZnO nanoparticles | Jiménez *et al.,* 2015 | 10.1071/CH14123 | Synergistic effects with metals |
| The synergetic antibacterial activity of Ag islands on ZnO (Ag/ZnO) heterostructure nanoparticles and its mode of action. | Zhang *et al.,* 2014 | 10.1016/j.jinorgbio.2013.10.004 | Synergistic effects with metals |
| Superior antibacterial activity of zinc oxide/graphene oxide composites originating from high zinc concentration localized around bacteria | Wang *et al.,* 2014 | 10.1021/am4053317 | Synergistic effects with other materials |

**Table S3.** Methodological quality and risk-of-bias assessment of *in vivo* studies using SYRCLE’s tool.

| **Domain** | **Sequence generation (Selection bias)** | **Baseline characteristics (Selection bias)** | **Allocation concealment (Selection bias)** | **Random housing (Performance bias)** | **Blinding (Performance bias)** | **Random outcome assessment (Detection bias)** | **Blinding (Detection bias)** | **Incomplete outcome data (Attrition bias)** | **Selective outcome reporting (Reporting bias)** | **Other sources of bias (Other)** | **Overall** |
| --- | --- | --- | --- | --- | --- | --- | --- | --- | --- | --- | --- |
| **Bahadori Zade et al., 2024** | Unclear | Low | Unclear | Unclear | Unclear | Unclear | Unclear | Low | Low | Low | Unclear |
| **Abdelghafar et al., 2022** | Unclear | Low | Unclear | Unclear | Unclear | Unclear | Unclear | Low | Low | Low | Unclear |
| **El-Telbany et al., 2022** | High | Low | Unclear | Unclear | Unclear | Unclear | Low | Low | Low | Low | Unclear |
| **Shoukani et al., 2024** | Unclear | Low | Unclear | Unclear | Unclear | Unclear | Unclear | Low | Low | Low | Unclear |
| **Saddik et al. (2022)** | Unclear | Low | Unclear | Unclear | Unclear | Unclear | Unclear | Low | Low | Low | Unclear |

**Table S4.** Main methods used to assess antimicrobial interaction and their interpretative limitations.

| **Method** | **What it evaluates** | **Strengths** | **Main limitations** | **Interpretation in this review** |
| --- | --- | --- | --- | --- |
| **Disk diffusion / inhibition zone** | Increase or reduction in bacterial growth around antimicrobial agents | Simple, low-cost, useful for initial screening | Does not quantify drug interaction; affected by diffusion, nanoparticle dispersion, medium composition, and compound solubility | Considered evidence of enhanced antimicrobial activity, but not sufficient alone to confirm synergy |
| **MIC/MBC reduction** | Reduction in the concentration required to inhibit or kill microorganisms | Quantitative and widely used in antimicrobial testing | MIC/MBC reduction may indicate potentiation or additivity, but does not by itself distinguish true synergy from enhanced activity | Interpreted as enhanced antimicrobial activity or potential synergistic behavior when not accompanied by FICI or time-kill data |
| **Checkerboard / FICI** | Interaction between two antimicrobial agents across concentration combinations | Provides quantitative interaction data; commonly used to classify synergy, additivity, indifference, or antagonism | Cutoff values may vary among studies; results depend on concentration range, strain, inoculum, and assay conditions | Considered stronger evidence of synergy when FICI ≤ 0.5 was reported |
| **Time-kill assay** | Dynamic reduction in viable bacterial counts over time | Captures bactericidal kinetics and temporal interaction between agents | More labor-intensive; less frequently performed; depends on selected time points and concentrations | Considered strong evidence of synergy when reported according to accepted log-reduction criteria |
| **Biofilm assays** | Inhibition, reduction, or eradication of biofilm formation | Clinically relevant for chronic and device-associated infections | Biofilm models are heterogeneous; antibiofilm improvement does not necessarily prove pharmacological synergy | Interpreted as antibiofilm enhancement unless combined with quantitative synergy criteria |
| **Statistical comparison alone** | Significant difference between isolated and combined treatments | Useful to support differences between groups | Statistical significance does not define the type of antimicrobial interaction | Considered supportive evidence only, not sufficient to confirm synergy |

**Abbreviations:** MIC: minimum inhibitory concentration; MBC: minimum bactericidal concentration; FICI: fractional inhibitory concentration index.
